# The role of government in helping SMEs to access finance: An evolutionary game modeling and simulation approach

**DOI:** 10.1371/journal.pone.0315941

**Published:** 2024-12-27

**Authors:** Zhu Mei, Jingjing Zhang, QiaoMei Zhou

**Affiliations:** 1 School of Management, Jiangsu University, Zhengjiang, Jiangsu Province, China; 2 School of Intellectual Property, Jiangsu University, Zhengjiang, Jiangsu Province, China; University of Almeria: Universidad de Almeria, SPAIN

## Abstract

**Purpose:**

This study aims to delineate the operating system of a strategic game model involving three core financial actors—government, banks, and guarantee institutions, with a focus on their collective impact on system evolution towards sustainable SME financing.

**Methodology:**

Utilizing numerical simulations informed by dynamic equation constraints and optimal equilibrium states, this paper abstracts the strategic behaviors of system constituents, constructing a game model to predict and analyze system evolution within various operational contexts.

**Results:**

The simulation experiments reveal the critical role of quality risk information and responsible actor behavior in maintaining low default rates and fostering a sustainable financial system. System trajectories under various scenarios highlight the fragility of the equilibrium and the necessity of concerted, strategic cooperation among all stakeholders.

**Conclusions:**

Findings underscore the importance of a cooperative, conscientious approach by government, banks, and guarantee institutions to ensure a robust and sustainable SME financing environment. The study advocates for strategic policy guidance, emphasizing the interconnectedness of institutional roles and their cumulative effect on system stability.

## 1. Introduction

Small and medium-sized enterprises (SMEs) face significant financing challenges due to a lack of adequate collateral and credit history, a common issue in both developed and developing countries. Particularly, with the ongoing changes in the global economic cycle and the risks of downturns, the financing pressure on SMEs is further intensified. To alleviate this predicament, various countries have formulated and implemented financing guarantee programs [1]. For instance, Italy has implemented a public guarantee scheme aimed at leveraging public credit to assist guarantee institutions, thereby enhancing the creditworthiness of SMEs to secure bank loans. In the United States, the Small Business Administration (SBA) launched the 7(a) Loan Program during the financial crisis, encouraging cooperation between the government and private investors to jointly invest in and provide loan guarantees for small businesses. In Japan, Credit Guarantee Corporations (CGCs) collaborate with banks to establish a comprehensive financing guarantee system to assist SMEs in obtaining financing [2].

These financing guarantee programs share commonalities in their construction logic, where the cooperation between banks and guarantors is the focal point of the implementation of guarantee plans in various countries, forming a "Bank + Guarantee Institution" cooperation model. The main role of the guarantor is to enhance the creditworthiness of SMEs and reduce the lending risk of banks through credit endorsement and sharing of compensation [3]. The implementation of the "Bank + Guarantee Institution" model has made an important contribution to helping SMEs obtain loans and promoting economic recovery. However, it also faces some common problems.

Firstly, there is an information asymmetry in the perception of risk among banks, guarantors, and SMEs. SMEs have the motive to conceal risks, while banks, as holders of financial discourse power, have the motive to shift risks to guarantors. If the guarantor, with limited capacity, cannot fully assess the risks, it will become the sole underwriter of risks. This leads to a situation where banks use whether a company has a guarantee as the basis for lending, and guarantors, finding it hard to bear the heavy responsibility of risk, become cautious in guaranteeing [4]. Ultimately, this results in an adverse locked situation where loan costs increase, the difficulty of loan issuance increases, and the volume of loan issuance decreases. Secondly, SMEs, due to the reduced loan risk with the help of guarantees, do not manage their finances prudently, leading to an increase in the risk of default in the opposite direction, making risk prevention itself fragile.

To address this, China has drawn on the experience of developed countries and its own national conditions to further introduce government participation, building upon the "Bank + Guarantee Institution" cooperation model. A "Government + Bank + Guarantee Institution" (GBGI) cooperation guarantee model has been established, specifically aimed at SMEs. Around this model, various provinces in China have carried out diverse practical explorations. Although there are slight differences in specific measures, the essence is the same: emphasizing the role and function of the government within the GBGI model. The attempt is to alleviate the "Bank-Guarantee" cooperation dilemma through government involvement, enhance creditworthiness, and more efficiently serve the financing needs of SMEs [5].

In guarantee programs worldwide, the presence of governments is ubiquitous. For instance, the Italian government’s "Fondo di Garanzia" program emphasizes the significant role of public credit in expanding the coverage of guarantees. The U.S. 7(a) Guarantee Program demonstrates the government’s key role in attracting private capital to enter the market for cooperative investment and guarantees [2]. The Japanese government has allocated funds to establish the Small and Medium Enterprise Finance Corporation, which serves as a credit supplement for CGCs, ensuring the proper functioning of the credit system. However, these government roles are more focused on external guidance and support for guarantee programs, attempting to use public credit as an auxiliary to the guarantee plans [11].

In contrast, China’s GBGI model is different, emphasizing the government’s deep involvement in the guarantee program, that is, in-depth intervention in various aspects such as risk review and risk assumption [4]. For example, in the practical exploration of the GBGI model in Shandong, China, the Shandong government acts as a co-insurer with the guarantee institution, providing post-event risk subsidies to the guarantee institution when compensation occurs. In the practical exploration of the GBGI model in Anhui, the Anhui government participates in the pre-loan selection and recommendation of SME clients, adopting a proportional risk-sharing approach to bear the financing risks of SMEs together with guarantee institutions and banks.

Due to the deep involvement of the government, the GBGI model appears to better manage credit risk and control default rates, but it also brings some negative impacts. For instance, in the GBGI practice in Anhui, it was found that over-reliance on government client screening can lead to a reduction in the initiative of banks and guarantee institutions in pre-loan risk control. Furthermore, indiscriminate risk subsidies and risk sharing may increase the default risk of SMEs, resulting in low system efficiency. The government faces a dilemma: if it participates less, intervenes less, and does not provide sufficient subsidies, it will hinder banks from expanding credit scale. However, if it deeply participates and provides high subsidies, it may lead to a lack of autonomy and enthusiasm in banks and guarantee institutions, and a relaxation of risk review. It is evident that how the government adopts appropriate measures for participation and finds the correct role positioning in the GBGI model is the key for the GBGI model to function truly. Exploring the correct role of the government and effective measures in the GBGI model also has significant practical significance and value.

Reviewing past research reveals that scholars have already conducted studies on the role and measures of the government in financing guarantees [6–9]. However, these literatures have only demonstrated the important role of government support in alleviating the financing difficulties of SMEs. Since past research has been limited to viewing the government as an auxiliary in financing guarantees, discussions on government measures have also been confined to the level of encouragement, guidance, and support. There has been a failure to explore how the government’s role and measures should change when it participates in financing guarantees. From the review of literature related to cooperation in financing guarantees, scholars have discussed the credit-enhancement mechanisms of bank and guarantee institution cooperation in financing [10–12], explored the credit-enhancing roles of fixed assets, digital technologies, new indicator systems, and risk-sharing in financing guarantees, emphasizing the importance of helping banks and guarantee institutions identify and share the risks of SMEs. Among these, risk-sharing is an important credit-enhancement instrument. Scholars have further discussed the risk-sharing ratio, risk-sharing measures, and so on [13–15], they argue that multi-party participation in risk-sharing is an effective way to expand financing, and the more parties involved, the more new technologies applied, the better the ability to identify the real risks of SMEs, reducing the asymmetry of risk information. These studies provide an important theoretical basis for this paper’s research, that is, in the GBGI model, the government, as a participant in financing guarantees, should also transform its role based on risk-sharing, further transforming to help banks and guarantee institutions better identify the real risks of SMEs. The measures it adopts should also strive to make the risk information blind spots between banks, guarantee institutions, and SMEs more transparent, reduce the asymmetry of risk information, mobilize the enthusiasm of banks and guarantee institutions for financing, and increase the credit of SMEs.Based on the aforementioned analysis, the purpose of this paper’s research is to:

1. Explore how the government, after its role transformation in the GBGI model, should act to incentivize banks and guarantee institutions to actively participate in the GBGI model and jointly identify the real risks of SMEs.
2. What factors can promote effective cooperation between the government, banks, and guarantee institutions to ensure the efficient and sustainable operation of the GBGI model?

By exploring these research objectives, this paper can fill the theoretical gap in the exploration of the government’s role in the transformation in financing guarantees, which is the theoretical value of this research. It can also provide useful policy recommendations for the sustainable development of the GBGI model. Based on this, the paper carries out the following research.

In the second section, the paper will conduct a detailed literature review to lay a theoretical foundation for this research and elucidate the theoretical gaps. In the third section, the paper will first systematically introduce the operating mechanism and interactions of the GBGI model; secondly, it will construct an evolutionary game model for the three parties involved, centered around the operating mechanism. In the fourth section, the paper will solve the constructed evolutionary game model, exploring the conditions for achieving an effective equilibrium in the GBGI model and deducing effective measures for the government. In the fifth section, the paper will set specific situational parameters and carry out scenario simulation experiments to explore the situational conditions for the efficient and sustainable operation of the GBGI model, thereby further demonstrating the effectiveness of government measures; finally, based on the simulation results, the paper will draw the research conclusions, present the research contributions, and propose future perspectives.

## 2. Literature review

### 2.1 Research on the mechanism of financing guarantee cooperation

SMEs consistently face challenges in financing. Firstly, the weak or unstructured accounting systems of SMEs affect the quality of financial information, making it difficult to convey good creditworthiness to banks [16]. Secondly, the risk of high default rates and low credit ratings also discourage banks from lending or result in higher interest rates [17]. Thirdly, the broad and substantial presence of SMEs, coupled with the opacity of risk information, increases the lending costs for banks [18]. These are significant factors affecting SMEs’ access to loans. To address these issues, since the early 20th century, countries including the United States [19], Japan [20], the Middle East and North Africa region [17], South Korea [21, 22], and China [23] have all implemented financing guarantee mechanisms. The aim is to use guarantees from guarantee institutions to help banks diversify lending risks and reduce lending costs, thereby increasing the financing success rate for SMEs.

Currently, both theory and practice have demonstrated that financing guarantee mechanisms can improve the financing conditions of SMEs in many countries, including both developed and developing nations [24]. Particularly during crises, such as during the COVID-19 pandemic, financing guarantee institutions have rescued numerous SMEs struggling for survival [25]. Despite significant differences among countries in loan pricing, guarantee ratios, risk-sharing ratios, and industry and regional restrictions [26], guarantee institutions indeed share the lending risks of banks and reduce information asymmetry and the collateral burden on SMEs, effectively helping SMEs to secure loan supply [16]. Among these, the cooperation model between banks and guarantee institutions, as an important approach to solving the problems of difficult and expensive financing for SMEs, has received widespread attention and research.

The working paper considers macroeconomic variables, policy variables, and the creditworthiness of SMEs as important factors in pricing guarantee fees. It also emphasizes the reduction of non-performing assets of guarantee institutions and the enhancement of the robustness and stability of the guarantee system. Farhad et al. (2019) emphasizes the formulation of reasonable guarantee plans and the use of advanced guarantee techniques to compensate for the information disadvantage of SMEs, thereby increasing the willingness of banks to lend [27].

Third-party guarantees are an effective way to enhance the creditworthiness of SMEs [11, 28] and can alleviate the "financial exclusion" of SMEs by banks [29]. However, guarantee institutions still face issues of insufficient credit enhancement, and the mortgage of fixed assets remains a key element in credit enhancement [11]. Risk-sharing, as an important credit-enhancing measure of guarantee institutions, helps to increase the motivation and efficiency of cooperation between banks and guarantee institutions, thereby better-assisting SMEs in financing. However, the current imbalance in the risk-reward ratio between banks and guarantee institutions severely hinders the further development of guarantee business [30]. Some scholars have used different methods to calculate an optimal risk-sharing ratio. Liang & Mei (2013), considering the practicality issues of applying mathematical models to calculate the risk-sharing ratio between banks and guarantee institutions, proposed the use of the Elman neural network model to study the risk-sharing ratio between banks and guarantee institutions [31]. Verma (2023) pointed out that technological innovation can better assess risks and adjust the risk-sharing ratio between guarantee institutions and banks [32]. Karima and Walid (2023) proposed a risk-sharing-based loan guarantee technology, covering potential losses through de facto equity participation agreements and reducing the risk of premature default [13].

However, banks and guarantee institutions, as profit-oriented enterprises, pursue the maximization of benefits. In risk-sharing, both parties are in a zero-sum game. Therefore, without reducing the probability of risk, both sides tend to lower their own risk-sharing ratio; on the contrary, under a given risk-sharing ratio, to avoid losses, they may raise the threshold for financing guarantees for SMEs, establish stricter screening conditions, thereby reducing risk. With the development of the financial market, to break out of the poor lock-in state of bank-guarantee cooperation, research has begun to expand to the participation of multiple entities, exploring how multiple entities can jointly act to form a credit-enhancing synergy [15, 33–35]. At the same time, with the rise of financial technology, technologies such as blockchain, big data, and artificial intelligence have been applied in the credit-enhancing mechanism, improving the accuracy and efficiency of risk assessment [36–38].

In summary, past research has demonstrated the positive role of financing guarantee cooperation mechanisms in supporting the financing of SMEs, but there are also issues with insufficient credit enhancement. Since risk-sharing is an effective way to expand financing, some studies, based on optimizing the existing risk-sharing ratios between banks and guarantee institutions, tend to explore the participation of multiple entities and the application of emerging technologies. They anticipate the role of identifying the real risks of SMEs and reducing the asymmetry of risk information. This indicates that the existing financing guarantee cooperation model still needs to introduce more themes and further optimize in terms of risk-sharing and credit enhancement.

### 2.2 Research on the role of government in SME financing

Small and medium-sized enterprises (SMEs) are characterized by their small scale, lack of collateral, and limited credit history, making it difficult for banks to assess the risks associated with these businesses, resulting in an information asymmetry in risk perception. Consequently, banks may adopt a higher ratio or refuse to lend to SMEs [39]. Monzur (2023) suggests that government intervention can help reduce the asymmetry in risk perception between banks and SMEs by increasing the supply of loanable funds to banks and assisting in the identification of creditworthy clients [40]. Crawford (2023) posits that government credit can regulate the allocation of bank financing, significantly promoting the technological innovation investment and output of SMEs [41]. Wilcox and Yasuda (2019) found that the Japanese government’s credit can serve as a complement to unsecured loans, alleviating the risk adversity faced by banks and making them more willing to lend to risky SMEs [42]. Government credit possesses a "national certification effect," and government guidance and supervision are beneficial in reducing the risks associated with technological innovation, thereby helping businesses to enhance their creditworthiness and alleviate financing constraints [6]. These studies have demonstrated that the government can act as a bridge, mitigating the information asymmetry between SMEs and banks, enhancing the creditworthiness of SMEs, and thereby increasing the willingness of banks to lend. That is, the government plays a crucial role in credit enhancement.

Among the various credit enhancement measures, the government’s risk-sharing is an important approach to improving the financing of small and medium-sized enterprises (SMEs) [23, 43, 44]. Yoshino and Farhad (2019) argue that policy-based guarantees can provide a proportional risk-sharing for bank loans to SMEs, which can significantly enhance the banks’ confidence in lending [16]. Xu et al. (2022) propose that the risk-sharing of policy-based guarantees has effectively promoted the compliance and financing guarantee cooperation of SMEs in the short term [45]. Countries such as Japan have established full policy-based guarantee programs, aiming to cover 100% of the default costs for SMEs, but also indicating that while such measures can increase the financing access of SMEs, they may also lead to moral hazard, causing banks to lack the incentive to assess and monitor the health of borrowers, thereby increasing the number of non-performing loans and reducing the productivity of public reserves [20, 46]. These studies suggest that risk-sharing is an important measure for the government to enhance the creditworthiness of SME financing, but indiscriminate risk-sharing may lead to adverse consequences.

Additionally, the government can also enhance the creditworthiness of SMEs and support their access to more financing through various guiding measures such as service innovation, system construction, subsidy policies, supervision and management, and financial assistance. For instance, Huang & Wang (2023) suggest that the government can encourage the increase of bank lending through technological services and adopt differentiated regulatory approaches for the cooperation between banks and fintech [47]. Crawford (2023) explores how the government can provide policy support for financing guarantees for SMEs [41]. Studies indicate a lack of synergy between direct financing and soft support, with soft support allowing SMEs to achieve higher success rates or enhance their ability to effectively deploy new funds. Some authors have explored the direct guiding effect of government funding [7], and Du et al. (2017) using a sample of 908 companies from 2000 to 2010, found that SMEs in areas with high levels of government intervention or with strong government backgrounds are more likely to improve credit and obtain loans [48]. Xiang and Worthington (2017) found that financial assistance from the government helps SMEs improve performance, surpassing the effects of traditional financing. It was also found that SMEs that have received government financial assistance are more likely to obtain bank financing in the future [49]. However, some scholars believe that while government financial support can help SMEs survive to some extent, it may not necessarily help them achieve higher growth, and it is necessary to combine government support services with financing [50]. In addition, Kshetri (2016) concludes that the main reason for the lack of credit among Chinese micro-enterprises is the lack of data, information, and capabilities in banks to obtain risk information about enterprises [51].

In summary, past research has demonstrated that the government plays a crucial role in enhancing the creditworthiness of small and medium-sized enterprises (SMEs) in financing, where risk-sharing is an important measure, but indiscriminate risk-sharing may lead to adverse outcomes. Additionally, the government can assist SMEs in obtaining financing through various supportive policies such as service innovation and financial support. However, these studies only discuss the government’s role in enhancing credit between banks and SMEs, and there is a lack of exploration into the specific roles and measures of the government’s deeper involvement. Moreover, previous research has largely regarded the government as an auxiliary to SME financing, with the measures discussed mainly staying at the level of encouragement, guidance, and support. At the same time, when discussing the credit-enhancing role of government risk-sharing, there is a greater emphasis on proving the positive effects of government risk-sharing, and there is a lack of discussion on how to better achieve risk-sharing.

Previous studies have provided an important theoretical foundation for this research, highlighting the positive role of the bank-guarantee cooperation mechanism in supporting the financing of small and medium-sized enterprises (SMEs). However, there is an issue of insufficient credit enhancement, and government participation can further reduce risks and expand the credit-enhancing effect. Yet, the government’s indiscriminate and singular involvement may not fully resolve the problem. Therefore, exploring how the government, as a participant in the financing guarantee within the GBGI model, should transform its role and what measures should be taken to truly fulfill the government’s function, can fill the theoretical gap in this area of research.

## 3. Methodology

Evolutionary game theory, which originated from biological evolutionary theory [52], centers on simulating the evolution of strategies in the process of natural selection. It posits that under conditions of bounded rationality and information, individuals optimize their strategies through imitation and learning. This theory emphasizes the dynamic learning and adaptation of strategies, differing from classical game theory which assumes complete rationality and information and is more aligned with real-world economic conditions. Two key concepts of evolutionary game theory are "evolutionarily stable strategy" and "replicator dynamics" [53]. An evolutionarily stable strategy describes a state where the dominant strategy of a population is resistant to new strategies, while replicator dynamics simulate the changes in the distribution of population strategies over time. If a strategy yields higher returns than the population average, it will be adopted by more individuals; otherwise, it will be eliminated.

In this study, evolutionary game theory helps to analyze the strategic choices and interactions among government, banks, and guarantee institutions in SME financing guarantees under conditions of bounded rationality and information, thereby providing insights into solving the financing challenges of SMEs. By simulating and analyzing these interactions, key factors in improving the efficiency of financing guarantees can be identified, offering theoretical support for policy-making and practical operations. The model presented in this paper abstracts from real-world entities while ensuring adherence to ethical standards set by institutional or national research committees as well as comparable ethical standards such as those outlined in the 1964 Helsinki Declaration and its subsequent amendments.

### 3.1 GBGI model mechanisms and principal strategy selection

The GBGI model is characterized by the tripartite participation and collaborative division of labor among the government, banks, and guarantee institutions, with the government’s role transitioning from external guidance to deep involvement. The model aims to reduce and share the default risk of SMEs through the participation and collaboration of the three main bodies, to expand the multiple of bank guarantee credit, and to efficiently and sustainably channel financial funds for the development of SMEs. This is also the overall goal of the GBGI model’s operation. Among them, expanding the multiple of bank guarantee credit and channeling financial funds for SMEs are the first important goals of the model’s operation. However, this goal is in contradiction with the profitability of finance. If banks are to expand the credit multiple, the default risk of the entire financing guarantee system must be effectively reduced to alleviate the risk control concerns of banks. For banks, the most direct way to reduce risk is to completely transfer the risk to guarantee institutions. Since banks hold the initiative in capital, they have the motive and ability to completely transfer risk, which can be manifested as banks only lending to enterprises with guarantees, that is, adopting a passive coping strategy. Guarantee institutions do not have the ability to bear all the risks. As self-responsible entities, guarantee institutions either choose to reduce guarantees to avoid the bottom line of risk or choose to completely transfer the risk to the government, that is, also adopting a passive coping strategy. At this time, the government will be in a dilemma. If it does not bear or bears less risk, it will hinder the guarantee of guarantee institutions and thus hinder the lending of banks. However, if it bears the risk, it will evolve into a risk bottom-line bearer. That is, the government’s single risk-sharing cannot break the dilemma, and the government’s blind participation in GBGI makes it difficult to achieve the goals of the GBGI model operation.

Therefore, the government’s unilateral risk-sharing measures are insufficient and a change of approach is necessary. According to the analysis in the previous introduction and literature review, the key to the successful operation of the GBGI model lies in the government’s transition from blind-guidance to strategic-guidance. This can stimulate the enthusiasm of banks and guarantee institutions, and leverage their respective strengths for deeper cooperation to identify the real risks of SMEs, thereby reducing the risk information asymmetry among the main bodies. Referring to the research by Wu et al. (2021), government agencies can link information data from various departments to help improve the digital footprint of SMEs and increase the transparency of risk information [54]. Thus, strategic participation measures include the strategic sharing of information to encourage pre-loan risk identification and customer screening; comprehensive assessment of the performance of responsibility by banks and guarantee institutions in the GBGI model, and differentiated risk-sharing. The government expects to mobilize the enthusiasm of banks through strategic-guidance, prompting banks to be willing to invest human, material, and financial resources to identify risks. At the same time, it is expected to mobilize the enthusiasm of guarantee institutions, prompting them to take their responsibilities seriously, actively cooperate with banks, and through risk identification and screening of SME customers, continuously optimize the guarantee threshold, ensure that applicants who meet the entry qualifications are fully guaranteed, and truly benefit the guarantee services to SMEs with development potential. Ultimately, the three parties enter into active cooperation, work together to break the risk information gap of SMEs, improve the operational efficiency of the GBGI model, and achieve operational goals.

**Fig 1:**
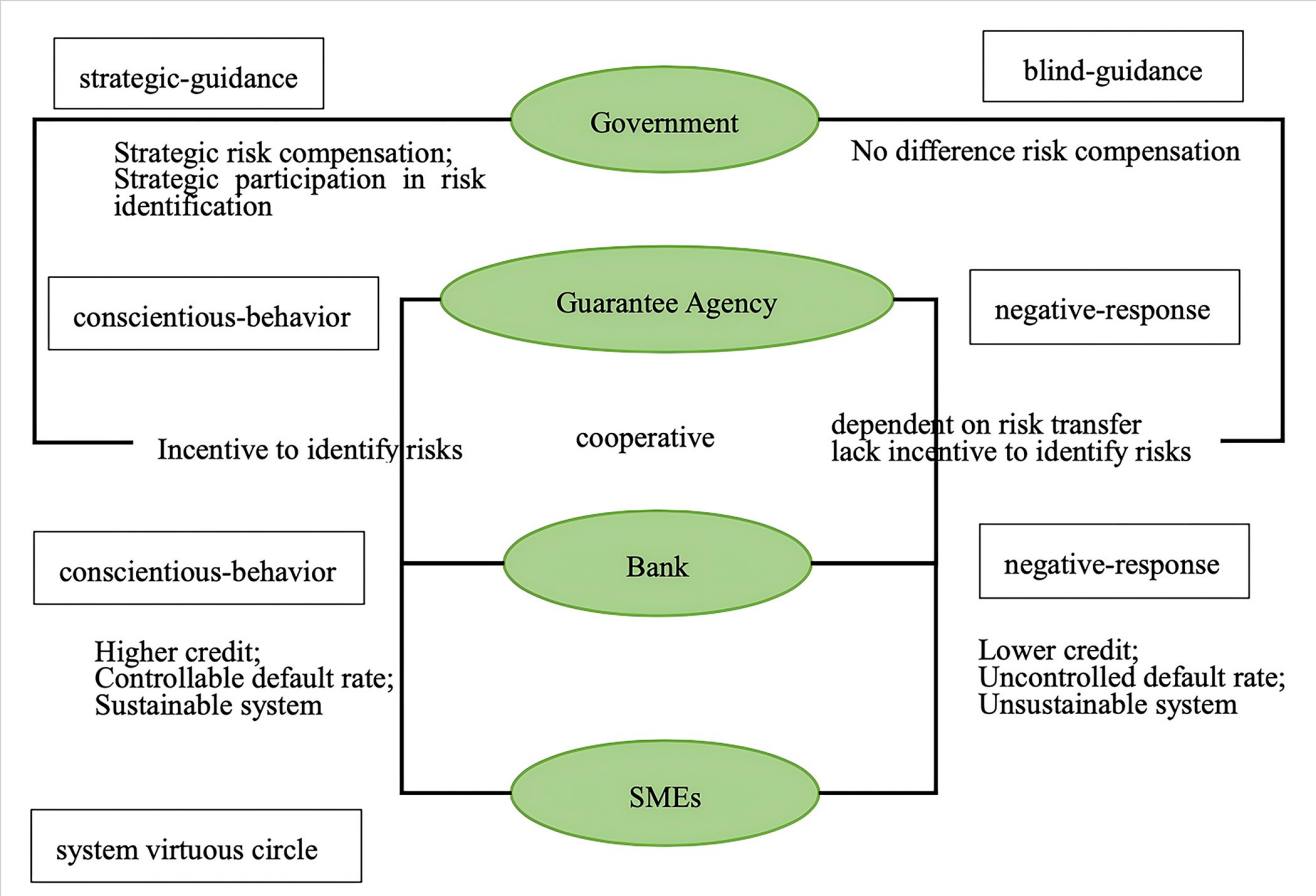
Conceptual model of agent strategy selection.

In summary, to facilitate effective cooperation between the government, banks, and guarantee institutions, and to ensure the efficient and sustainable operation of the GBGI model, it is necessary to use the government as a fulcrum to mobilize the enthusiasm of banks and guarantee institutions. The key lies in identifying the real risks of SMEs and selecting those with potential to provide financial support. However, due to the differences in the value attributes and goals of each entity, there is a natural tendency to choose passive coping strategies, which can lead the GBGI model into a suboptimal state. Therefore, based on clarifying the strategic choices and interactions of the three parties, this paper needs to further construct a game model to find equilibrium solutions that promote banks and guarantee institutions to actively fulfill their responsibilities and break the system’s suboptimal lock. Subsequently, it is necessary to identify effective strategies for government participation in GBGI. The conceptual model of the strategic choices of the government, banks, and guarantee institutions in the GBGI model is shown in Fig 1.

### 3.2 Game model establishment

Based on the analysis of the operating mechanism in the previous section, this paper further refines the behavioral strategies of the main bodies within the system and constructs a game model for the three participants: the government, banks, and guarantee institutions. By analyzing system evolution trends, it determines when institutional goals are achieved, and explores effective governmental guiding measures. The government strategy is summarized as "strategic-guidance" or "blind-guidance", the bank strategy as "conscientious-behavior" or "negative-response", and the strategy of guarantee institutions as "conscientious-behavior" or "negative-response ". This provides a theoretical basis for a government’s combinatorial strategy.

The government’s strategy of "blind-guidance" means it provides only a certain amount of risk compensation according to the system requirements of the model, such as proportional default and non-differential risk subsidies to banks or guarantee institutions [55]. "Strategic-guidance" behavior refers to (1) strategic participation in ex-post risk-sharing. The government bears a proportional share of the risk of default, comprehensively evaluates the performance status of cooperative banks and guarantees institutions in the model, and provides differential risk subsidies according to their performance status; (2) strategic participation in pre-lending risk identification and customer screening. The government searches for and inspects the real status of SMEs and shares information with banks and guarantee agencies [56]. This information should help them identify the risks to SME customers, screen out SMEs with development potential, and guarantee their financing. When the government takes strategic guidance actions, there is an additional cost; however, it will also gain public recognition, which will lead to a higher social reputation [45].

The bank’s “negative-response” behavior strategy refers to the institutional requirement of the “government-bank-guarantee” model to provide loans to SMEs that have obtained guarantees and share the risk proportionally. Banks eschew the expenditure in conducting risk assessments for SMEs. The loan origination rate under this strategy is lower and may be exposed to some policy losses, while the overall vicarious risk of the system may increase because of a lack of careful risk identification [57]. The “Conscientious-behavior” strategy refers to not only proportional risk sharing but also active participation in risk identification and customer screening of SME customers. For example, we can develop financial products to support small businesses and screen and identify the financing risks and development potential of our customers. When banks choose to take their responsibilities seriously, they must pay more for risk identification and screening costs [58].

The “negative-response” behavior strategy of the guarantee agency means that it reviews the SME customer’s qualification and gives the guarantee according to policy requirements. Under this strategy, the guarantor will only review the client responsively. The guarantee business volume is small, the risk of repayment is high, and a blind guarantee results in a penalty from the government [59]. The “Conscientious-behavior” strategy refers to proportional risk sharing as well as active risk identification and screening of SME customers. For example, based on the written review, the guarantee agency further develops financial products to support small businesses and identifies customer risks and development potential. The guarantee agency will continue to optimize the guarantee threshold and guarantee that all applicants meet the entry qualifications to benefit SMEs with potential. This strategy requires more risk identification and screening costs for institutions [60, 61].

## 4. Analysis of evolutionary game model

### 4.1 Basic assumptions of the model

It is assumed that the government, banks, and guarantee institutions are all finite rational decision makers with limited information. It is difficult for each game player to make an optimal strategic choice in one event, and they need to gradually optimize their behavior by imitating and learning from each other in the process of the game.

Simplifying the interference of other factors, it is assumed that the default rate in this system is influenced only by the quality of risk information and the performance status of banks and guarantors. *V*_*i*_ is used to indicate the average default rate of SME customers in the system, and *infi* indicates the quality of risk information obtained by banks and guarantee institutions about their SME customers. In the case of government strategic guidance, it will search for and examine customer information and share information with banks and guarantee institutions. The quality of the risk information at this point is expressed as *inf*_*1*_.

In the case of government blind guidance, the quality of the obtained risk information is expressed as *inf*_*2*_ *(inf*_*1*_*>inf*_*2*_*)*. We use *α* to denote the probability that, in a natural state, the SME does not use funds for a specific development project after receiving the loan and is unable to repay the loan as promised when it is due. We assume that banks and guarantee institutions can reduce certain default risks, denoted by *β* and *γ (β<α*, *γ<α)*, respectively. The abilities of banks and guarantee institutions to perform their duties are *ζ*_*1*_ and *ζ*_*2*_ *(ζ*_*1*_*<1*,*ζ*_*2*_*<1*,*ζ*_*1*_*+ζ*_*2*_*<1)*_,_ respectively. The better the ability to fulfill the responsibility, the higher the quality of risk information and the greater the risk reduction, that is, *β* and *γ* will become larger, as *β = ζ*_*1*_^*2*^*·α·inf*_*i*_, *γ = ζ*_*2*_^*2*^*·α·inf*_*i*_. When the government is strategically guiding and both banks and guarantors are serious about their responsibilities, the default rate is *V*_*l*_ *= α-(ζ*_*1*_*+ζ*_*2*_*)*^*2*^*·α·inf*_*1*_. When the government is guiding strategically and only banks are serious about their responsibilities, the default rate is *V*_*2*_ *= α-ζ*_*1*_^*2*^*·α·inf*_*i*_,*(i = 1)*. When the government is guiding strategically and only guarantors are serious about their responsibilities, the default rate is *V*_*3*_ *= α-ζ*_*2*_^*2*^*·α·inf*_*i*_,*(i = 1)*. When the government is blindly guiding and both banks and guarantors are serious about their responsibilities, the default rate is *V*_*5*_ *= α-(ζ*_*1*_*+ζ*_*2*_*)*^*2*^*·α·inf*_*2*_. When the government is blindly guiding and only banks are serious about their responsibilities, the default rate is *V*_*6*_ *= α-ζ*_*1*_^*2*^*·α·inf*_*i*_,*(i = 2)*. When the government is guiding blindly and only guarantors are serious about their responsibilities, the default rate is *V*_*7*_ *= α-ζ*_*2*_^*2*^*·α·inf*_*i*_,*(i = 2)*. When neither banks nor guarantors are serious about their responsibilities, the default rate is the natural default rate of the system, as V_4_ = α.

*n*_*a*_*(a = 0*,*1*,*2)* is used to indicate the credit multiplier of the bank, which is determined by the bank, but is subject to the government and guarantee agencies. If the government guides strategically and banks and guarantee institutions are serious about their responsibilities, they can jointly design more new financing products to identify SME default risks based on higher-quality risk information. It can reduce systemic risks and screen out more potential SMEs, and banks will increase the guarantee multiplier represented by *n*_*1*_. If the government guides blindly but banks and guarantee institutions are serious about their responsibilities, they will also jointly design new financing products to identify SME default risk. The systemic risk default rate increases because the overall risk information quality decreases, and banks reduce the guarantee multiplier, represented by *n*_*2*_. When the guarantee agency has a negative response, the bank lacks a reliable guarantee and, based on the trade-off between risk and benefit, maintains the minimum guarantee multiplier requirement set by the system, denoted as *n*_*0*_. If banks are passive, they will not respond positively, even if the government guides strategically or the guarantee agency seriously fulfills its responsibility to provide more reliable guaranteed loan customers for banks, and the multiplier is maintained at *n*_*0*_, *n*_*1*_*>n*_*2*_*>n*_*0*_.

Banks and guarantee institutions choose to take their responsibilities seriously, which means they will work hard to reduce the risk of SME financing and expand the multiplier for SME financing. Assuming that these efforts incur a corresponding cost, the total cost can be expressed as *C*_*s*_. If both the bank and guarantor are serious about their responsibilities, they will cooperate, and the costs can be borne by the bank and guarantor. Let *λ*_*i*_ *(i = 0*,*1*,*2)*be the proportion of costs borne by the bank and *μ*_*j*_ *(j = 0*,*1*,*2)*the percentage of costs borne by the guarantee agency, as *λ*_*i*_*+μ*_*j*_ *= 1*. When only the bank chooses responsibility, *i = 1*, *λ*_*1*_ *= 1; j = 0*, *μ*_*0*_ *= 0*. When only the guaranteed agency chooses to take its responsibilities seriously, *i = 0*, *λ*_*0*_ *= 0; j = 1*, *μ*_*1*_ *= 1*. When banks and guarantee institutions are both serious about fulfilling their responsibilities, cooperation arises between them; then, *i = 2*, *λ*_*2*_*>0; j = 2*, *μ*_*2*_*>0; λ*_*2*_*+μ*_*2*_ *= 1*.

### 4.2 Profit and loss variables for each subject

#### (1) Profit and loss variables that affect government decisions

Let the risk fund established by the government be *K* and the credit multiplier be *n*_*a*_ *(a = 0*,*1*,*2)*. Then, the total amount of loans available to the SME is *n*_*a*_*k*. The return coefficient of the increase in local tax revenue when SMEs obtain a loan and use it for production is denoted by *Q*. The total return was *Qn*_*a*_*k*. The cost of blind guidance is represented as *C*_*g1*_, including non-discriminatory risk subsidies to banks or guarantee institutions. The cost of strategic guidance is represented as *C*_*g2*_, including the cost of searching and assessing the risk information and sharing the risk information of the SMEs to participate in the GBGI financing project, the cost of policy subsidies (such as guaranteed rate subsidy) for SMEs that pay on time, the cost of supervision to monitor the performance of banks and guarantee institutions, and the cost of incentives for banks and guarantee institutions that perform their duties. Owing to the inclusive financial nature of SME financing, it is assumed that all additional benefits, such as reputation and social benefits gained when the government guides strategically, can be quantified as *H*, and *A*_*1*_ is the percentage of default in place of payment borne by the government.

#### (2) Profit and loss variables affecting bank decisions

Banks earn interest income by providing loans to SMEs. Let *R*_*1*_ be the benchmark lending rate, and let the bank earn revenue from *R*_*1*_*n*_*a*_*k*. It is assumed that, in addition to the interest income, the bank will receive some additional benefits if it fulfills its responsibilities well, denoted as *Z*_*b*_. This includes local government incentives (e.g., tax exemptions, special subsidies, and industry incentives) and hidden benefits (e.g., social reputation, long-term benefits for quality customers, and potential development benefits). Banks that take their responsibilities seriously will have a risk identification cost (*λ*_*i*_*C*_*s*_) and an evaluation cost incurred in reviewing the guarantor (*C*_*k*_). If the bank is passive, it does not have to pay the aforementioned performance costs, but it will not receive additional benefits (*Z*_*b*_). When the government is actively guiding, the negative attitude of banks and guarantee institutions will affect their reputation and relationship with the government, thus impacting the development of other potential businesses. Let the penalty cost be *C*_*f1*_ and *A*_*2*_ be the percentage of compensation borne by the bank.

#### (3) Profit and loss variables affecting guarantor decision making

The guarantee agency earns profits by providing guarantees to the SMEs. Assuming that the guarantee rate is *R*_*2*_, then the revenue obtained from the guarantee is *R*_*2*_*n*_*a*_*k*. If the agency accepts its responsibilities well, it receives additional benefits when the government guides strategically, denoted as *Z*_*g*_. This includes rewards (such as tax exemptions, special subsidies, and industry awards) and hidden benefits (such as social reputation, long-term benefit for quality customers, and potential development benefit) received from government recognition for good performance of responsibilities. When the bank also takes its responsibilities seriously, it will gain long-term benefits w (business growth benefit, reputation benefit, etc.) due to cooperation between the two parties. Fulfilling the responsibility costs a certain amount of money *μ*_*j*_*C*_*s*_ *(j = 1*,*2)*, which is not incurred when the guarantee agency is passive and negative. Meanwhile, when the government is actively guiding, the negative attitude of banks and guarantee institutions will affect their reputation and relationship with the government, thus impacting the development of other potential businesses. Let the penalty cost be *C*_*f2*_ and *A*_*3*_ be the percentage of compensation borne by the bank.

### 4.3 Building of revenue function

When the government chooses strategic guidance, the bank chooses conscientious performance and the guarantee agency chooses conscientious performance, which is defined as *1*. When the government chooses blind guidance, the bank chooses a negative response and the guarantee agency chooses a negative response, which is defined as *0*. The payment matrices for the government, bank, and guarantee agency are shown in Table 1.

**Table 1:** Behavioral strategy combination and benefit matrix for government, bank, and guarantee agency.

Strategies combination	Government	Bank	Guarantee Agency
(1,1,1)	(1-V_1_)Qn_1_k+H-C_g2_-V_1_n_1_kA_1_	(1-V_1_)R_1_n_1_k+Z_b_-λ_2_C_s_-C_k_-V_1_n_1_KA_2_	(1-V_1_)R_2_n_1_k+Z_g_+w-μ_2_C_s_-V_1_n_1_KA_3_
(1,1,0)	(1-V_2_)Qn_0_k+H-C_g2_-V_2_n_0_kA_1_	(1-V_2_)R_1_n_0_k+Z_b_-λ_1_C_s_-C_k_-V_2_n_0_KA_2_	(1-V_2_)R_2_n_0_k-V_2_n_0_KA_3_-C_f2_
(1,0,1)	(1-V_3_)Qn_0_k+H-C_g2_-V_3_n_0_kA_1_	(1-V_3_)R_1_n_0_k-V_3_n_0_KA_2_-C_f1_	(1-V_3_)R_2_n_0_k+Z_g_-μ_1_C_s_-V_3_n_0_KA_3_
(1,0,0)	(1-V_4_)Qn_0_k+H-C_g2_-V_4_n_0_kA_1_	(1-V_4_)R_1_n_0_k-V_4_n_0_KA_2_-C_f1_	(1-V_4_)R_2_n_0_k-V_4_n_0_KA_3_-C_f2_
(0,1,1)	(1-V_5_)Qn_2_k-C_g1_-V_5_n_2_kA_1_	(1-V_5_)R_1_n_2_k+Z_b_-λ_2_C_s_-C_k_-V_5_n_1_KA_2_	(1-V_5_)R_2_n_2_k+Z_g_w-μ_2_C_s_-V_5_n_2_KA_3_
(0,1,0)	(1-V_6_)Qn_0_k-C_g1_-V_6_n_0_kA_1_	(1-V_6_)R_1_n_0_k+Z_b_-λ_1_C_s_-C_k_-V_6_n_0_KA_2_	(1-V_6_)R_2_n_0_k+Z_g_-V_6_n_0_KA_3_
(0,0,1)	(1-V_7_)Qn_0_k-C_g1_-V_7_n_0_kA_1_	(1-V_7_)R_1_n_0_k+Z_b_-V_7_n_0_KA_2_	(1-V_7_)R_2_n_0_k+Z_g_-μ_1_C_s_-V_7_n_0_KA_3_
(0,0,0)	(1-V_4_)Qn_0_k-C_g1_-V_4_n_0_kA_1_	(1-V_4_)R_1_n_0_k+Z_b_-V_4_n_0_KA_2_	(1-V_4_)R_2_n_0_k+Z_g_-V_4_n_0_KA_3_

U_11_ is the expected return of the government choosing the strategic guidance strategy, U_12_ is the expected return of the government choosing the blind guidance strategy, and U_1_ is the average expected return of the government. Accordingly

U_11_ = yz((1-V_1_)Qn_1_k+H-C_g2_-V_1_n_1_kA_1_)+y(1-z)((1-V_2_)Qn_0_k+H-C_g2_-V_2_n_0_kA_1_)+(1-y)z((1-V_3_)Qn_0_k+H-C_g2_-V_3_n_0_kA_1_)+(1-y)(1-z)((1-V_4_)Qn_0_k+H-C_g2_-V_4_n_0_kA_1_)

U_12_ = yz((1-V_5_)Qn_2_k-C_g1_-V_5_n_2_kA_1_)+y(1-z)((1-V_6_)Qn_0_k-C_g1_-V_6_n_0_kA_1_)+(1-y)z((1-V_7_)Qn_0_k-C_g1_-V_7_n_0_kA_1_)+(1-y)(1-z)((1-V_4_)Qn_0_k-C_g1_-V_4_n_0_kA_1_)

U_1_ = xU_11_+(1-x)U_12_

U_21_ represents the expected returns of the bank choosing the conscientious-performance strategy, U_22_ is the expected return of the bank choosing the negative-response strategy, and U_2_ is the average expected return of the bank. Thus

U_21_ = xz((1-V_1_)R_1_n_1_k+Z_b_-λ_2_C_s_-C_k_-V_1_n_1_KA_2_)+x(1-z)((1-V_2_)R_1_n_0_k+Z_b_-λ_1_C_s_-C_k_-V_2_n_0_KA_2_)+(1-x)z((1-V_5_)R_1_n_2_k+Z_b_-λ_2_C_s_-C_k_-V_5_n_1_KA_2_)+(1-x)(1-z)((1-V_6_)R_1_n_0_k+Z_b_-λ_1_C_s_-C_k_-V_6_n_0_KA_2_)

U_22_ = xz((1-V_3_)R_1_n_0_k-V_3_n_0_KA_2_-C_f1_)+x(1-z)((1-V_4_)R_1_n_0_k-V_4_n_0_KA_2_-C_f1_)+(1-x)z((1-V_7_)R_1_n_0_k+Z_b_-V_7_n_0_KA_2_)+(1-x)(1-z)((1-V_4_)R_1_n_0_k+Z_b_-V_4_n_0_KA_2_)

U_2_ = yU_21_+(1-y)U_22_

where U_31_ represents the expected returns of the guarantee agency choosing the conscientious-performance strategy, U_32_ is the expected return of the guarantee agency choosing the negative-response strategy, and U_3_ is the average expected return of the guarantee agency. Here

U_31_ = xy((1-V_1_)R_2_n_1_k+Z_g_+w-μ_2_C_s_-V_1_n_1_KA_3_)+x(1-y)((1-V_3_)R_2_n_0_k+Z_g_-μ_1_C_s_-V_3_n_0_KA_3_)+(1-x)y((1-V_5_)R_2_n_2_k+Z_g_w-μ_2_C_s_-V_5_n_2_KA_3_)+(1-x)(1-y)((1-V_7_)R_2_n_0_k+Z_g_-μ_1_C_s_-V_7_n_0_KA_3_)

U_32_ = xy((1-V_2_)R_2_n_0_k-V_2_n_0_KA_3_-C_f2_)+x(1-y)((1-V_4_)R_2_n_0_k-V_4_n_0_KA_3_-C_f2_)+(1-x)y((1-V_6_)R_2_n_0_k+Z_g_-V_6_n_0_KA_3_)+(1-x)(1-y)((1-V_4_)R_2_n_0_k+Z_g_-V_4_n_0_KA_3_)

U_3_ = zU_31_+(1-z)U_32_

### 4.4 Analysis of evolutionary stability strategy using replication dynamic equation

#### (1) Progressive stability analysis of government

The replication dynamic equation for government with a strategic guidance policy is as follows:

F(x) = dx/dt = x(U_11_-U_1_) = x(1-x)(U_11_-U_12_) = x(1-x)[C_g1_-C_g2_+H+y(V_6_-V_2_)(n_0_kA_1_+Qn_0_k)+yz[(Qn_1_K-Qn_2_k)-(V_1_n_1_KA_1_+QV_1_n_1_K)+V_5_(n_2_KA_1_+Qn_2_K)+(V_7_-V_3_)(n_0_KA_1_+Qn_0_K)+(V_2_-V_6_)(n_0_KA_1_+Qn_0_K)+(V_3_-V_7_)(n_0_KA_1_+Qn_0_K)]]

According to the stability theorem of the replication dynamic equation, when F(p0) = 0 and F’(p)|_p = p0_< 0, p = p0 is a stable strategy.

① If C_g1_-C_g2_+H+y(V_6_-V_2_)(n_0_kA_1_+Qn_0_k)+yz[(Qn_1_K-Qn_2_k)-(V_1_n_1_KA_1_+QV_1_n_1_K)+V_5_(n_2_KA_1_+Qn_2_K)+(V_7_-V_3_)(n_0_KA_1_+Qn_0_K)+(V_2_-V_6_)(n_0_KA_1_+Qn_0_K)+(V_3_-V_7_)(n_0_KA_1_+Qn_0_K)] = 0, then *F(x) = 0*, which means that all levels are stable; that is, the ratio of strategy selection in this case does not change with time.

② If C_g1_-C_g2_+H+y(V_6_-V_2_)(n_0_kA_1_+Qn_0_k)+yz[(Qn_1_K-Qn_2_k)-(V_1_n_1_KA_1_+QV_1_n_1_K)+V_5_(n_2_KA_1_+Qn_2_K)+(V_7_-V_3_)(n_0_KA_1_+Qn_0_K)+(V_2_-V_6_)(n_0_KA_1_+Qn_0_K)+(V_3_-V_7_)(n_0_KA_1_+Qn_0_K)]≠0, let *F(x) = 0*to obtain *x = 0*,*x = 1*,which are two stable points. The derivative of F(x) is as follows:.

F’(x) = (1-2x)[C_g1_-C_g2_+H+y(V_6_-V_2_)(n_0_kA_1_+Qn_0_k)+yz[(Qn_1_K-Qn_2_k)-(V_1_n_1_KA_1_+QV_1_n_1_K)+V_5_(n_2_KA_1_+Qn_2_K)+(V_7_-V_3_)(n_0_KA_1_+Qn_0_K)+(V_2_-V_6_)(n_0_KA_1_+Qn_0_K)+(V_3_-V_7_)(n_0_KA_1_+Qn_0_K)]] The two situations are discussed below:

When

C_g1_-C_g2_+H+y(V_6_-V_2_)(n_0_kA_1_+Qn_0_k)+yz[(Qn_1_K-Qn_2_k)-(V_1_n_1_KA_1_+QV_1_n_1_K)+V_5_(n_2_KA_1_+Qn_2_K)+(V_7_-V_3_)(n_0_KA_1_+Qn_0_K)+(V_2_-V_6_)(n_0_KA_1_+Qn_0_K)+(V_3_-V_7_)(n_0_KA_1_+Qn_0_K)] <0, F’(0)<0, F’(1)>0, and thus, x = 0 is the equilibrium point, indicating that if the benefits of the strategic guidance strategy are less than the costs, then the blind-guidance strategy is the evolutionary stability strategy for the government.

When

C_g1_-C_g2_+H+y(V_6_-V_2_)(n_0_kA_1_+Qn_0_k)+yz[(Qn_1_K-Qn_2_k)-(V_1_n_1_KA_1_+QV_1_n_1_K)+V_5_(n_2_KA_1_+Qn_2_K)+(V_7_-V_3_)(n_0_KA_1_+Qn_0_K)+(V_2_-V_6_)(n_0_KA_1_+Qn_0_K)+(V_3_-V_7_)(n_0_KA_1_+Qn_0_K)] >0, F’(0)>0, F’(1)<0, and thus, x = 1 is the equilibrium point and the strategic guidance strategy is the evolutionary stability strategy for the government.

#### (2) Progressive stability analysis of banks

The replication dynamic equation for a bank choosing the conscientious-performance strategy is as follows:

F(y) = dy/dt = y(U_21_-U_2_) = y(1-y)(U_21_-U_22_) = y(1-y)[-C_k_+kR_1_(n_0_(V_4_+V_6_(-1+x)-V_2_x)+(n_2_(-1+V_5_)(x-1)-n_1_(V_1_-1)x+n_0_(V_6_+V_7_-V_4_-1+(V_2_+V_3_)x-(V_6_+V_7_)x))z)+A_2_k(n_1_(V_5_(x-1)-V_1_x)z+n_0_(V_4_+V_6_(x-1)-V_2_x+(V_6_-V_4_+V_7_+(V_2_+V_3_) x-(V_6_+V_7_)x)z))+x(C_f1_+Z_b_)+C_s_(z-1)λ_1_-λ_2_C_s_z]

①If

-C_k_+kR_1_(n_0_(V_4_+V_6_(-1+x)-V_2_x)+(n_2_(-1+V_5_)(x-1)-n_1_(V_1_-1)x+n_0_(V_6_+V_7_-V_4_-1+(V_2_+V_3_)x-(V_6_+V_7_)x))z)+A_2_k(n_1_(V_5_(x-1)-V_1_x)z+n_0_(V_4_+V_6_(x-1)-V_2_x+(V_6_-V_4_+V_7_+(V_2_+V_3_)

x-(V_6_+V_7_)x)z))+x(C_f1_+Z_b_)+C_s_(z-1)λ_1_-λ_2_C_s_z = 0, then F(y) = 0, which means all levels are steady states, that is, the strategy selection ratio does not change with time.

②If

-C_k_+kR_1_(n_0_(V_4_+V_6_(-1+x)-V_2_x)+(n_2_(-1+V_5_)(x-1)-n_1_(V_1_-1)x+n_0_(V_6_+V_7_-V_4_-1+(V_2_+V_3_)x-(V_6_+V_7_)x))z)+A_2_k(n_1_(V_5_(x-1)-V_1_x)z+n_0_(V_4_+V_6_(x-1)-V_2_x+(V_6_-V_4_+V_7_+(V_2_+V_3_)

x-(V_6_+V_7_)x)z))+x(C_f1_+Z_b_)+C_s_(z-1)λ_1_-λ_2_C_s_z≠0, let F(y) = 0 to obtain y = 0, y = 1, which are two stable points. The derivative of F(y) is as follows:

F’(y) = (1-2y)[-C_k_+kR_1_(n_0_(V_4_+V_6_(-1+x)-V_2_x)+(n_2_(-1+V_5_)(x-1)-n_1_(V_1_-1)x+n_0_(V_6_+V_7_-V_4_-1+(V_2_+V_3_)x-(V_6_+V_7_)x))z)+A_2_k(n_1_(V_5_(x-1)-V_1_x)z+n_0_(V_4_+V_6_(x-1)-V_2_x+(V_6_-V_4_+V_7_+(V_2_+V_3_)

x-(V_6_+V_7_)x)z))+x(C_f1_+Z_b_)+C_s_(z-1)λ_1_-λ_2_C_s_z]. The two cases are discussed below.

When

− C_k_+kR_1_(n_0_(V_4_+V_6_(-1+x)-V_2_x)+(n_2_(-1+V_5_)(x-1)-n_1_(V_1_-1)x+n_0_(V_6_+V_7_-V_4_-1+(V_2_+V_3_)

x-(V_6_+V_7_)x))z)+A_2_k(n_1_(V_5_(x-1)-V_1_x)z+n_0_(V_4_+V_6_(x-1)-V_2_x+(V_6_-V_4_+V_7_+(V_2_+V_3_)

x-(V_6_+V_7_)x)z))+x(C_f1_+Z_b_)+C_s_(z-1)λ_1_-λ_2_C_s_z<0, F’(0)<0, F’(1)>0, y = 0 is the equilibrium point, indicating that the benefits of the conscientious-performance strategy

When − C_k_+kR_1_(n_0_(V_4_+V_6_(-1+x)-V_2_x)+(n_2_(-1+V_5_)(x-1)-n_1_(V_1_-1)x+n_0_(V_6_+V_7_-V_4_-1+(V_2_+V_3_)

x-(V_6_+V_7_)x))z)+A_2_k(n_1_(V_5_(x-1)-V_1_x)z+n_0_(V_4_+V_6_(x-1)-V_2_x+(V_6_-V_4_+V_7_+(V_2_+V_3_)

x-(V_6_+V_7_)x)z))+x(C_f1_+Z_b_)+C_s_(z-1)λ_1_-λ_2_C_s_z>0, F’(0)>0, F’(1)<0, y = 1 is the equilibrium point, and the conscientious-performance strategy is the evolutionary stability strategy.

#### (3) Progressive stability analysis of the guarantee agency

The replication dynamic equation for guaranteeing that the agency chooses the conscientious-performance strategy is as follows:

F(z) = dz/dt = z(U_31_-U_3_) = z(1-z)(U_31_-U_32_) = z(1-z)[xC_f2_+wxy+kR_2_((n_2_(-1+V_5_)(x-1)-n_1_(V_1_-1)x)y+n_0_(V_4_-V_7_-V_3_x+V_7_x+(-1-V_4_+V_6_+V_7_+(V_2_+V_3_)x-(V_6_+V_7_)x)y))+A_3_k(n_2_V_5_(-1+x)y-n_1_V_1_xy+n_0_(V_4_+V_7_(-1+x)-V_3_x+(-V_4_+V_6_+V_7_+(V_2_+V_3_)x-(V_6_+V_7_)x)y))+xZ_g_-yZ_g_+wyZ_g_+xyZ_g_-wxyZ_g_-C_s_μ_1_+C_s_yμ_1_-C_s_yμ_2_]

①If

xC_f2_+wxy+kR_2_((n_2_(-1+V_5_)(x-1)-n_1_(V_1_-1)x)y+n_0_(V_4_-V_7_-V_3_x+V_7_x+(-1-V_4_+V_6_+V_7_+(V_2_+V_3_)x-(V_6_+V_7_)x)y))+A_3_k(n_2_V_5_(-1+x)y-n_1_V_1_xy+n_0_(V_4_+V_7_(-1+x)-V_3_x+(-V_4_+V_6_+V_7_+(V_2_+V_3_)x-(V_6_+V_7_)x)y))+xZ_g_-yZ_g_+wyZ_g_+xyZ_g_-wxyZ_g_-C_s_μ_1_+C_s_yμ_1_-C_s_yμ_2_ = 0, F(z) = 0), which implies that all levels are stable. In other words, the strategy selection ratio does not change with time.

②If

xC_f2_+wxy+kR_2_((n_2_(-1+V_5_)(x-1)-n_1_(V_1_-1)x)y+n_0_(V_4_-V_7_-V_3_x+V_7_x+(-1-V_4_+V_6_+V_7_+(V_2_+V_3_)x-(V_6_+V_7_)x)y))+A_3_k(n_2_V_5_(-1+x)y-n_1_V_1_xy+n_0_(V_4_+V_7_(-1+x)-V_3_x+(-V_4_+V_6_+V_7_+(V_2_+V_3_)x-(V_6_+V_7_)x)y))+xZ_g_-yZ_g_+wyZ_g_+xyZ_g_-wxyZ_g_-C_s_μ_1_+C_s_yμ_1_-C_s_yμ_2_≠0. Let F(z) = 0 to obtain z = 0 and z = 1. The derivative of F(y) is as follows:.

F’(z) = (1-2z)[xC_f2_+wxy+kR_2_((n_2_(-1+V_5_)(x-1)-n_1_(V_1_-1)x)y+n_0_(V_4_-V_7_-V_3_x+V_7_x+(-1-V_4_+V_6_+V_7_+(V_2_+V_3_)x-(V_6_+V_7_)x)y))+A_3_k(n_2_V_5_(-1+x)y-n_1_V_1_xy+n_0_(V_4_+V_7_(-1+x)-V_3_x+(-V_4_+V_6_+V_7_+(V_2_+V_3_)x-(V_6_+V_7_)x)y))+xZ_g_-yZ_g_+wyZ_g_+xyZ_g_-wxyZ_g_-C_s_μ_1_+C_s_yμ_1_-C_s_yμ_2_], two cases are discussed below:

When xC_f2_+wxy+kR_2_((n_2_(-1+V_5_)(x-1)-n_1_(V_1_-1)x)y+n_0_(V_4_-V_7_-V_3_x+V_7_x+(-1-V_4_+V_6_+V_7_+(V_2_+V_3_)x-(V_6_+V_7_)x)y))+A_3_k(n_2_V_5_(-1+x)y-n_1_V_1_xy+n_0_(V_4_+V_7_(-1+x)-V_3_x+(-V_4_+V_6_+V_7_+(V_2_+V_3_)x-(V_6_+V_7_)x)y))+xZ_g_-yZ_g_+wyZ_g_+xyZ_g_-wxyZ_g_-C_s_μ_1_+C_s_yμ_1_-C_s_yμ_2_<0, F’(0)<0, F’(1)>0, and thus, z = 0 is the equilibrium point, indicating that if the benefits of conscientious-performance strategy are less than the costs, then the negative-response strategy is the evolutionary stability strategy for the guarantee agency.

When xC_f2_+wxy+kR_2_((n_2_(-1+V_5_)(x-1)-n_1_(V_1_-1)x)y+n_0_(V_4_-V_7_-V_3_x+V_7_x+(-1-V_4_+V_6_+V_7_+(V_2_+V_3_)x-(V_6_+V_7_)x)y))+A_3_k(n_2_V_5_(-1+x)y-n_1_V_1_xy+n_0_(V_4_+V_7_(-1+x)-V_3_x+(-V_4_+V_6_+V_7_+(V_2_+V_3_)x-(V_6_+V_7_)x)y))+xZ_g_-yZ_g_+wyZ_g_+xyZ_g_-wxyZ_g_-C_s_μ_1_+C_s_yμ_1_-C_s_yμ_2_>0, F’(0)>0, F’(1)<0, and thus, z = 1 is the equilibrium point and the conscientious-performance strategy is the evolutionary stability strategy for the guarantee agency.

#### (4) Stability analysis of game model

The stability strategy combination obtained through asymptotic stability analysis is not necessarily a stable strategy for a game system. The equilibrium point of strategy combination is as follows:(0,0,0)、(1,0,0)、(0,0,1)、(1,0,1)、(0,1,0)、(1,1,0)、(0,1,1)、(1,1,1). According to the Lyapunov stability theorem, if all the eigenvalues of the Jacobian are negative, then the equilibrium point is a stable point. The stability analysis of the game system is presented in Table 2.

**Table 2:** The stability analysis of the game system.

Equilibrium point	λ_1_	λ_2_	λ_3_
(1,1,1)	-C_g1_+C_g2_-H+k(-n_1_Q+n_1_(A_1_+Q)V_1_+n_2_(Q-(A_1_+Q)V_5_))	C_k_-C_f1_+k(-n_1_R_1_+n_1_(A_2_+R_1_)V_1_+n_0_(R_1_-(A_2_+R_1_)V_3_))-Z_b_+C_s_ λ_2_	-C_f2_+k(-n_1_R_2_+n_1_(A_3_+R_2_)V_1_+n_0_(R_2_-(A_3_+R_2_)V_2_))-w-Z_g_+C_s_μ_2_
(1,1,0)	-C_g1_+C_g2_-H+kn_0_(A_1_+Q) (V_2_-V_6_)	C_k_-C_f1_+kn_0_(A_2_+R_1_)(V_2_-V_4_)-Z_b_+C_s_ λ_1_	C_f2_+k(-n_0_R_2_+n_1_(R_2_-(A_3_+R_2_)V_1_)+n_0_(A_3_+R_2_)V_2_)+w+Z_g_-C_s_μ_2_
(1,0,1)	-C_g1_+C_g2_-H+kn_0_(A_1_+Q)(V_3_-V_7_)	-C_k_+C_f1_+k(-n_0_R_1_+n_1_(R_1_-(A_2_+R_1_)V_1_)+n_0_ (A_2_+R_1_)V_3_)+Z_b_-C_s_λ_2_	-C_f2_+kn_0_(A_3_+R_2_)(V_3_-V_4_)-Z_g_+C_s_μ_1_
(1,0,0)	-C_g1_+C_g2_-H	-C_k_+C_f1_+kn_0_(A_2_+R_1_)(-V_2_+V_4_)+Z_b_-C_s_ λ_1_	C_f2_+kn_0_(A_3_+R_2_)(-V_3_+V_4_)+Z_g_-C_s_μ_1_
(0,1,1)	C_g1_-C_g2_+H+k((n_1_-n_2_)Q-n_1_(A_1_+Q)V_1_+n_2_(A_1_+Q)V_5_)	C_k_+k(R_1_(n_0_+n_2_(-1+V_5_))+A_2_n_1_V_5_-n_0_(A_2_+R_1_)V_7_)+C_s_λ_2_	k(-n_2_R_2_+n_2_(A_3_+R_2_)V_5_+n_0_(R_2_-(A_3_+R_2_)V_6_))+Z_g_-wZ_g_+C_s_μ_2_
(0,1,0)	C_g1_-C_g2_+H-kn_0_(A_1_+Q) (V_2_-V_6_)	C_k_-n_0_k(A_2_+_R1_) (V_4_-V_6_)+C_s_λ_1_	k(-n_0_R_2_+n_2_(R_2_-(A_3_+R_2_)V_5_)+n_0_(A_3_+R_2_) V_6_)+(w-1) Z_g_-C_s_μ_2_
(0,0,1)	C_g1_-C_g2_+H-kn_0_(A_1_+Q)(V_3_-V_7_)	-C_k_-k(R_1_(n_0_+n_2_(-1+V_5_))+A_2_n_1_V_5_)+n_0_k(A_2_+R_1_)V_7_-C_s_λ_2_	-kn_0_(A_3_+R_2_)(V_4_-V_7_)+C_s_μ_1_
(0,0,0)	C_g1_-C_g2_+H	-C_k_+kn_0_(A_2_+R_1_)(V_4_-V_6_)-C_s_λ_1_	kn_0_(A_3_+R_2_)(V_4_-V_7_)-C_s_μ_1_

The equilibrium points must be discussed according to the results of the stability analysis. To make the cooperation strategy of agents evolve towards the ideal direction of (1,1,1), the following conditions must be met.

- [(1-V_1_)Qn_1_k-(1-V_5_)Qn_2_k]-[V_5_n_2_kA_1_-V_1_n_1_kA_1_]-(C_g1_-C_g2_)-H<0
- [(1-V_1_)R_1_n_1_k-(1-V_3_)R_1_n_0_k]-[V_3_n_0_kA_2_-V_1_n_1_kA_2_]-[C_f1_-C_k_-C_s_ λ_2_]-Z_b_<0
- [(1-V_1_)R_2_n_1_k-(1-V_2_)R_2_n_0_k]-[V_2_n_0_kA_3_-V_1_n_1_kA_3_]-(C_f2_-C_s_μ_2_)-Z_g_-w<0

This means that the government should ensure strategic guidance yields more gain and less loss than blind guidance. The bank should aim for conscientious performance to exceed the benefits and reduce the losses of a negative response. Likewise, the guarantee agency should confirm serious performance brings more benefits and less default loss than a negative response. In summary, factors such as risk information quality, default risk, loss, reward benefits, penalty costs, and performance cost-sharing ratio all influence participant behavior, thereby changing the overall system context. It’s crucial to study this selective influence based on the stability of one participant for insight into the system’s evolution.

## 5 Scenario simulation experiment and result analysis

### 5.1 Parameter value setting for the initial context

According to the constraints of replication dynamic equations and optimal equilibrium (1,1,1), the interactive behavior evolution process of the local government, banks, and guarantee institutions was numerically simulated and analyzed using MATLAB software. x0, y0, and z0 represent the initial proportions when the government selects “strategic guidance,” banks select “conscientious performance,” and guarantee institutions select “conscientious performance.” Its value is assumed to be 0.2, which implies that the initial state is (0.2, 0.2, 0.2). The initial time was 0, and the end time of the evolution was 10.

The parameter settings in the simulation are based on literature and practical experience, such as the practical context of the GBGI model operation in Anhui Province, if banks and guarantee institutions are serious about fulfilling their responsibilities, they will share costs, reduce the default rate, and jointly improve the guaranteed credit ratio. Banks and guarantee institutions can obtain government incentives for the conscientious performance of their responsibilities and guarantee that institutions will be recognized by banks and obtain long-term benefits for the conscientious performance of their responsibilities. When banks and guarantee agencies are passive and negative, the government punishes them.

**Fig 2:**
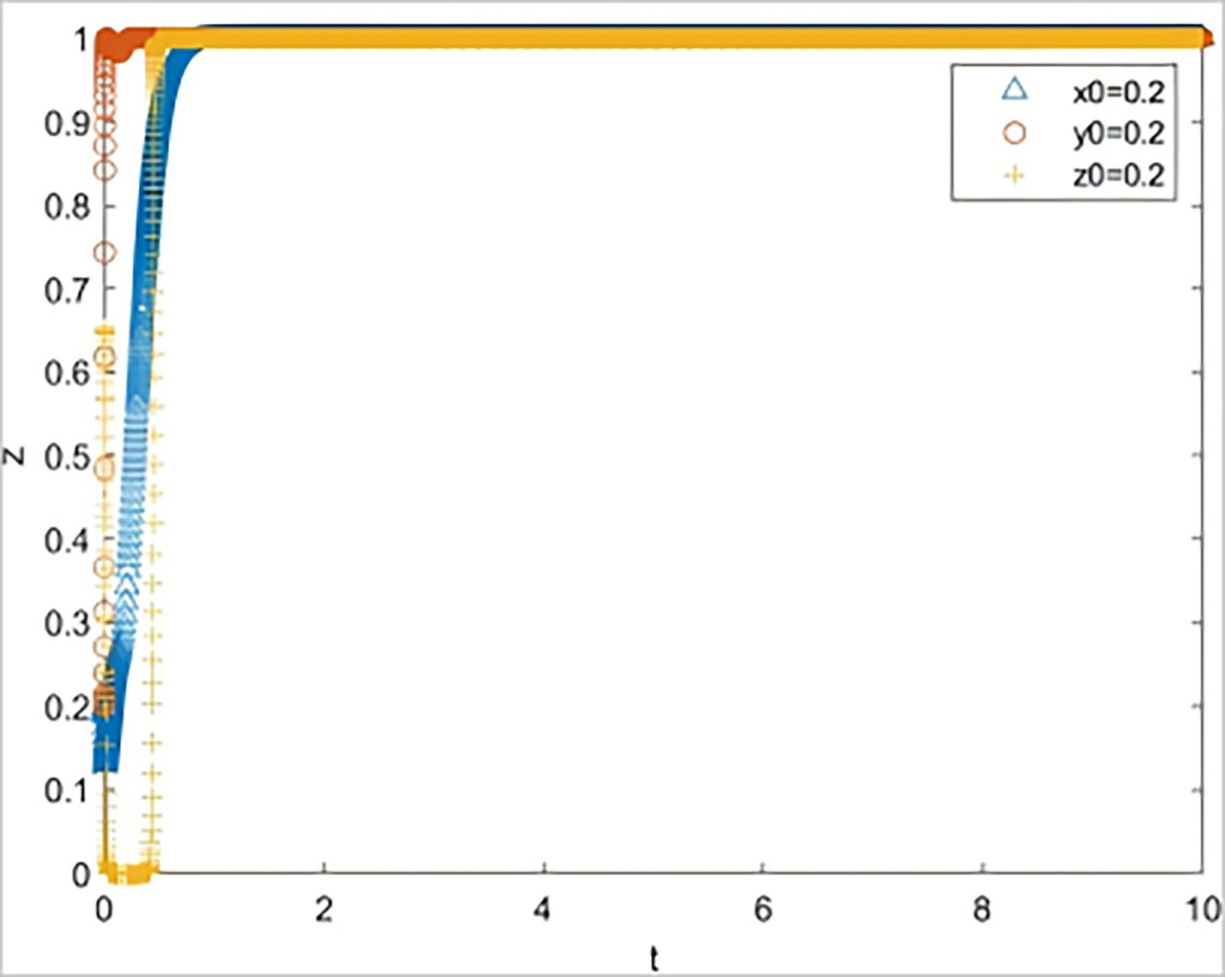
System simulation diagram under initial parameter setting.

Further referencing documents such as the "Guiding Opinions on Accelerating the Construction of the Policy-based Financing Guarantee System" issued by the General Office of the People’s Government of Anhui Province, specific parameter settings are made in accordance with the principle of equilibrium equations. The initial values are as follows: H = 300; Q = 2; C_g1_ = 200; C_g2_ = 200; k = 10000; R_1_ = 5.5%; R_2_ = 3%; n_0_ = 1.5; n_1_ = 5; n_2_ = 2.5; A_1_ = 0.2; A_2_ = 0.2; A_3_ = 0.6; V_1_ = 0.017; V_2_ = 0.057; V_3_ = 0.057; V_4_ = 0.3; V_5_ = 0.25; V_6_ = 0.25; V_7_ = 0.25; λ_1_ = 1; λ_2_ = 0.3; μ_1_ = 1; μ_2_ = 0.7; C_s_ = 200; C_k_ = 50; C_f1_ = 30; C_f2_ = 30; Z_b_ = 40; Z_g_ = 40; w = 40;

The system simulation results under the initial parameter settings are shown in Fig 2. At this point, the system is in the ideal state of (1,1,1), which is used as a reference to compare the changes in each parameter.

### 5.2 Multi-scenario simulation

#### (1) Scenario simulation of changes in the quality of the risk information provided by the government

Ideally, the effect of the government’s strategic guidance is good, which means that the risk information provided is true, reliable, practical, effective, and of high quality (*inf*_*1*_ *= 1*. Banks and guarantee institutions can accurately identify, screen, prevent, and control the financial risks by performing their responsibilities, thereby reducing the financial default rate of the entire system. If the two subjects cooperate, the default rate is controlled as *V*_*l*_ *= α-(ζ*_*1*_*+ζ*_*2*_*)*^*2*^*·α·inf*_*1*_ *= 0*.*017*; if the bank takes responsibility, the default rate is controlled as *V*_*2*_ *= α-β = α-ζ*_*1*_^*2*^*·α·inf*_*1*_ *= 0*.*057*; if the guarantee agency alone takes responsibility, the default rate is controlled as *V*_*3*_ *= α-γ = α-ζ*_*2*_^*2*^*·α·inf*_*1*_ *= 0*.*057*; if neither subject takes responsibility, the default rate is bound by nature as *V*_*4*_ *= α = 0*.*3*. The simulation results are shown in the figure, where the three subjects move to an ideal state of cooperation (strategic guidance, conscientious performance, and conscientious performance). The system enters a virtuous cycle of “conscientious performance by each subject—low default rate—further conscientious performance by each subject.”

① The simulation of subject strategy selection changes when the risk information quality decreases from inf_1_ = 1 to inf_1_ = 0.8.

**Fig 3:**
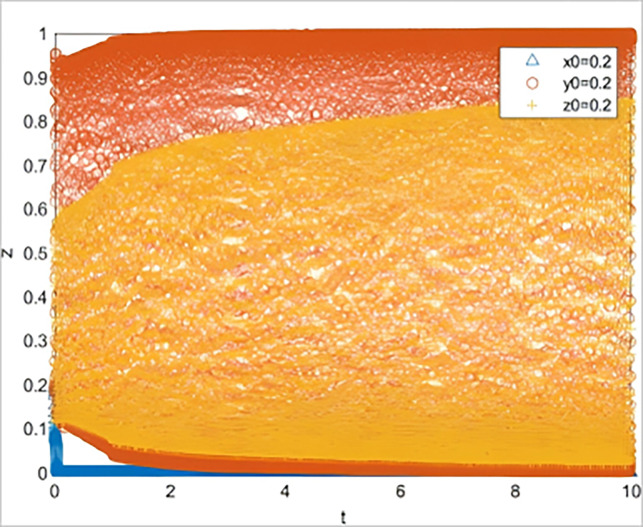
The impact on system evolution when the quality of risk information is reduced to 0.8.

Other conditions being equal, it is assumed that the quality of risk information shared by the government with banks and guarantors is slightly reduced, as *inf*_*1*_ *= 0*.*8*. Then, the default rate changes depending on whether banks and guarantors fulfill their responsibilities well, as *V*_*l*_ *= 0*.*07、V*_*2 =*_ *0*.*1、V*_*3*_ *= 0*.*1*. The simulation results are shown in Fig 3 The three subjects were in an unstable state.

② The simulation of subject strategy selection changed when the risk information quality decreased from inf_1_ = 1 to inf_1_ = 0.4.

**Fig 4:**
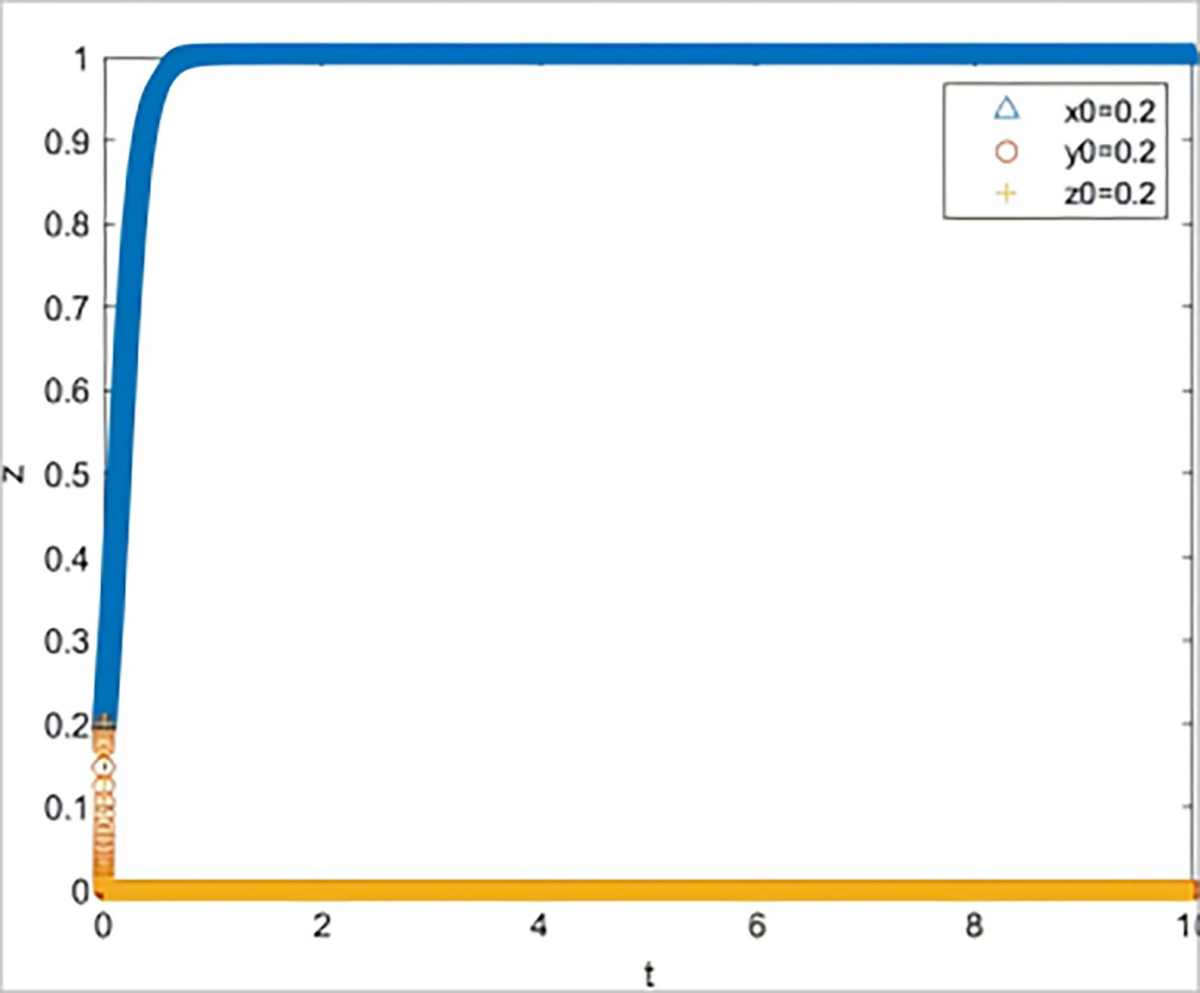
The impact on system evolution when the quality of risk information is reduced to 0.4.

Other conditions remain unchanged, and it is assumed that the quality of the SME risk information shared by the government with banks and guarantors is significantly lower in terms of reliability, usefulness, and validity *(inf*_*1*_ *= 0*.*4)*. At this point, the default rate changes depending on whether banks and guarantors fulfill their responsibilities well (*V*_*l*_ *= 0*.*18*, *V*_*2*_ *= 0*.*2*, *V*_*3*_ *= 0*.*2*, *V*_*4*_ *= 0*.*3*). The simulation results are shown in Fig 4, where all three subjects (strategic guidance, negative response, and negative response) head to a non-ideal state.

The results of two scenario simulation experiments indicate that under the strategic guidance of the government, even a slight decline in the quality of risk information provided for SMEs can lead to a significant increase in the default rate, thereby affecting the subsequent behavioral decisions of all parties involved. The quality of risk information is the key to triggering a chain reaction in the system. When the quality of risk information is low, banks and guarantee institutions, even if they fulfill their responsibilities diligently, find it difficult to reduce the default rate. If the default rate remains at a high level over the long term, it implies that the three parties involved must bear a higher risk when participating in GBGI projects. Without corresponding compensatory returns, the GBGI financing guarantee system will fall into a vicious cycle of "passive coping by all parties—sustained high default rate—further passive coping by all parties." Therefore, the provision of high-quality risk information by the government is a key element in breaking the vicious cycle of the system.

#### (2) Simulation of scenarios with the government’s differential rewards and punishments

When strategically guiding, the government supervises the behavior of banks and guarantee institutions and provides differentiated rewards and punishments. For example, it offers subsidy-like rewards to banks and guarantee institutions that fulfill their responsibilities diligently and imposes penalties on those that adopt a passive approach. The guidance cost C_g2_ paid by the government includes the reward costs, equivalent to the Z_b_ and Z_g_ rewards received by banks and guarantee institutions that fulfill their responsibilities diligently. When banks and guarantee institutions jointly choose to fulfill their responsibilities diligently, the guarantee institution will gain long-term benefits w due to its cooperation with the bank. In the initial ideal scenario, Z_b_ = 40, Z_g_ = 40, w = 40, C_f1_ = 30, C_f2_ = 30. The three main bodies move towards the ideal cooperative state of (strategic guidance, diligent responsibility, diligent responsibility), and the system enters a virtuous cycle of "all parties diligently fulfilling their responsibilities—receiving rewards—all parties further diligently fulfilling their responsibilities."

Scenario 2.1: Simulation of the situation where the bank’s reward value remains unchanged, and the government reduces the reward value for the guarantee institution.

**Fig 5:**
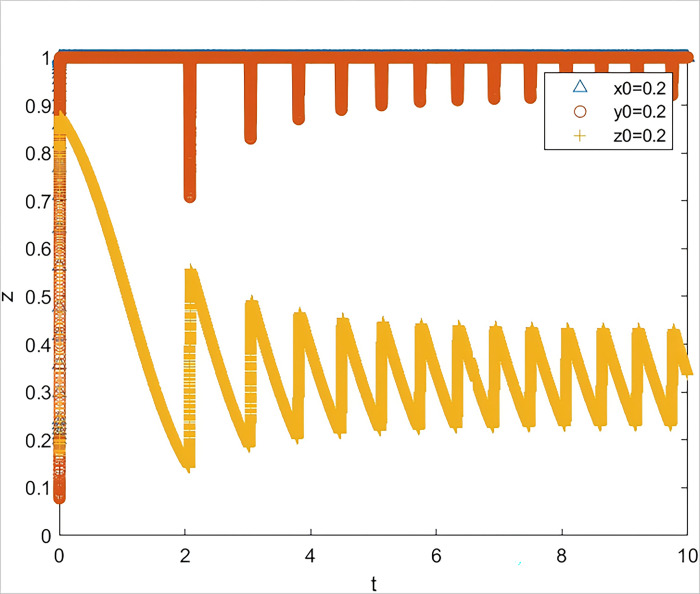
The impact on system evolution when the guarantor incentive is reduced (*Z*_*g*_ *= 30*).

Other scenario conditions remain unchanged, assuming Z_b_ remains at 40, w = 40, and Z_b_ is reduced to 30. The simulation results, as shown in Fig 5, indicate that the system has entered an unstable state.

**Fig 6:**
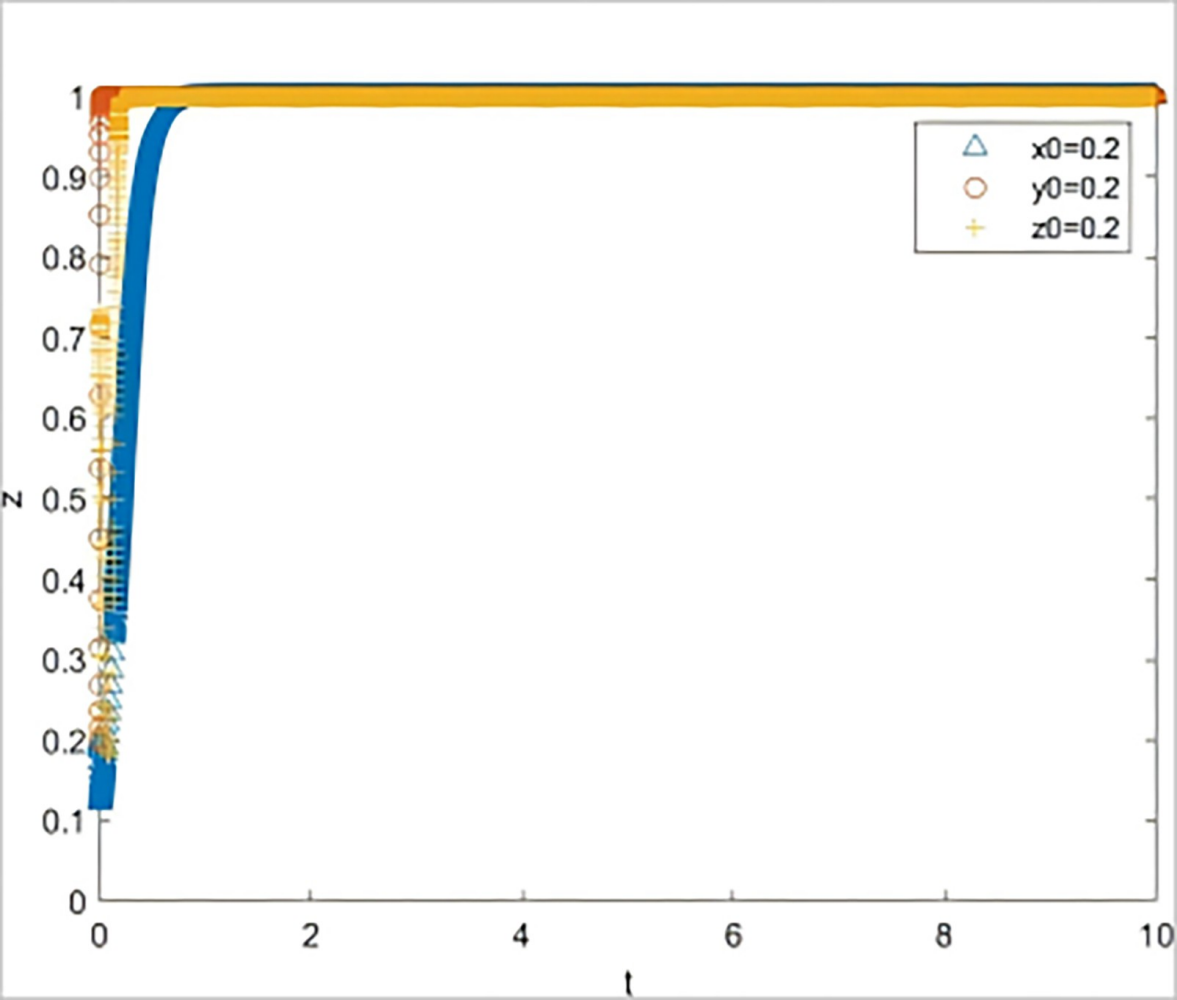
The impact on system evolution when the guarantor incentive is reduced (*Z*_*g*_ *= 30*) and the long-term gain is increased (*w = 50*).

This demonstrates that guarantee institutions are sensitive to the government’s rewards, which can trigger changes in the behavior of banks and the government, leading to system instability. Further simulation shows that if the long-term benefit w is increased to 50, at this point, the simulation results, as depicted in Fig 6, show that the system returns to stability, and the three parties re-enter the ideal state.

Scenario 2.2: Simulation of the situation where the guarantee institution’s reward value remains unchanged, and the government reduces the reward value for the bank.

**Fig 7:**
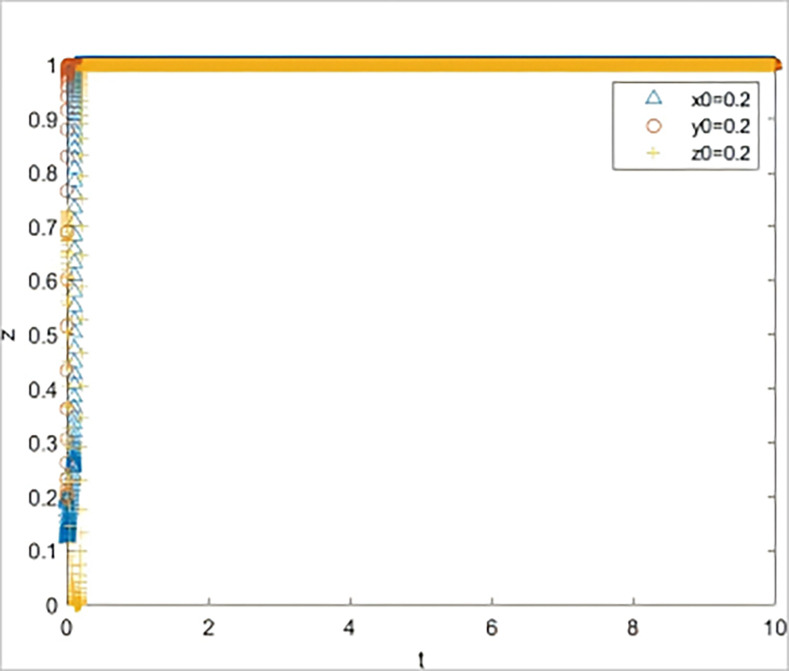
The impact on system evolution when the bank incentive is reduced (*Z*_*b*_ *= 10*).

With all other scenario conditions held constant, it is assumed that Z_g_ remains at 40, while Z_b_ is reduced to 10. The simulation reveals no change in the system (as shown in Fig 7).

This indicates that compared to guarantee institutions, banks are not sensitive to the government’s rewards. Consequently, the government can save on these rewards, thereby reducing its own reward costs.

Scenario 2.3: Simulation of the situation where the bank’s punishment intensity remains unchanged, and the government reduces the punishment intensity for the guarantee institution.

**Fig 8:**
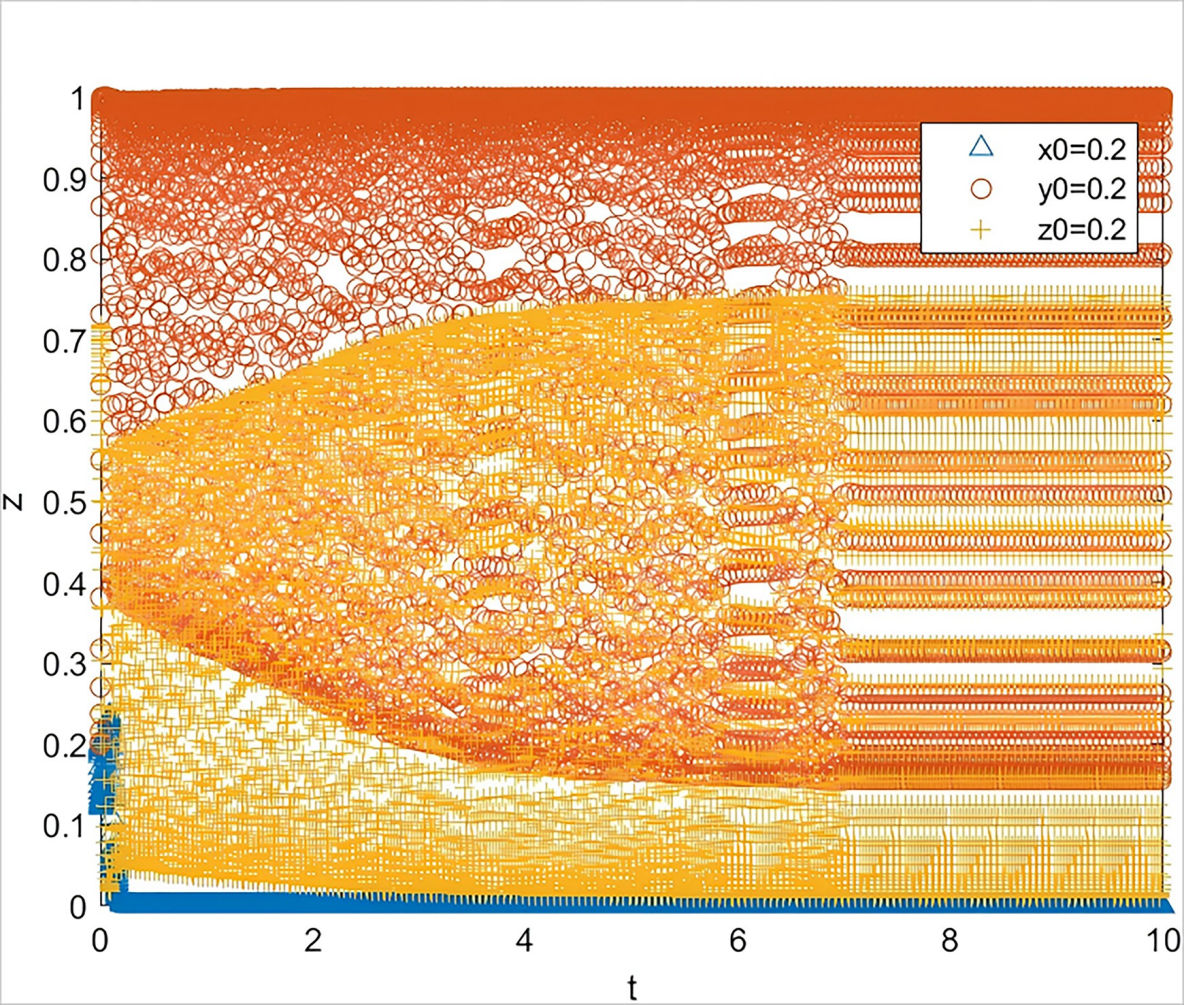
The impact on system evolution when the guarantor penalty is reduced (*C*_*f2*_ *= 10*).

With all other scenario conditions held constant, it is assumed that C_f1_ remains at 30, while C_f2_ is reduced to 20. The simulation results, as shown in Fig 8, indicate that the system has entered an unstable state.

**Fig 9:**
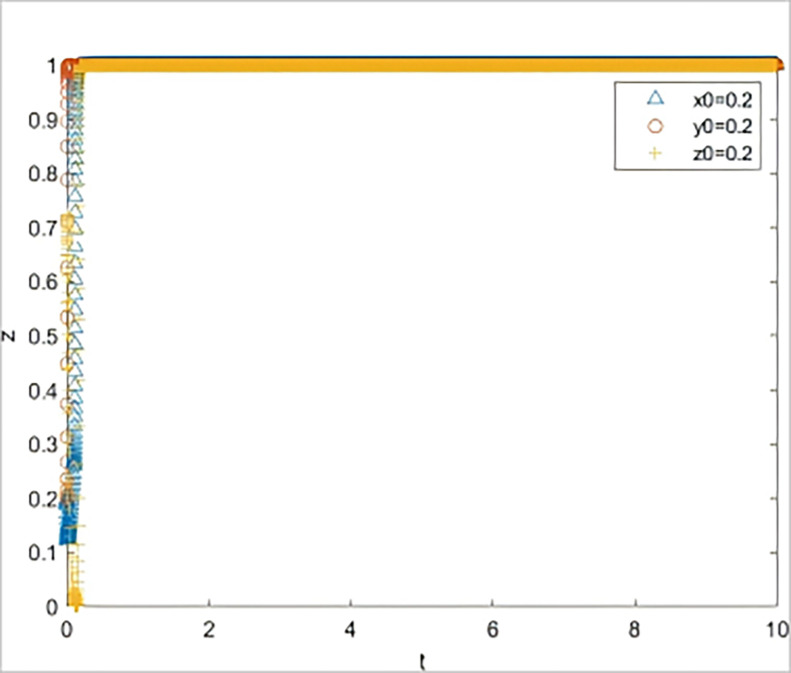
The impact on system evolution when the guarantor incentive is reduced (*C*_*f2*_ *= 10*) and the *Z*_*g*_ is increased to *50*.

This demonstrates that guarantee institutions are sensitive to the government’s punishments, and if the punishment for not fulfilling responsibilities diligently is reduced, guarantee institutions will enter a state of not fulfilling responsibilities diligently. This, in turn, triggers changes in the behavior decisions of the government and banks, leading to system instability. Further simulation shows that if the reward for guarantee institutions that fulfill their responsibilities diligently is increased, with Z_g_ rising to 50 (to ensure that the government’s total reward cost remains unchanged, the reward for banks is simultaneously reduced, with Z_b_ falling to 30), the simulation results, as depicted in Fig 9, show that the system returns to stability. This indicates that widening the gap between rewards and punishments for diligent and passive behavior can guide guarantee institutions back to the ideal state and restore system stability.

Scenario 2.4: Simulation of the situation where the guarantee institution’s punishment intensity remains unchanged, and the government reduces the punishment intensity for the bank.

**Fig 10:**
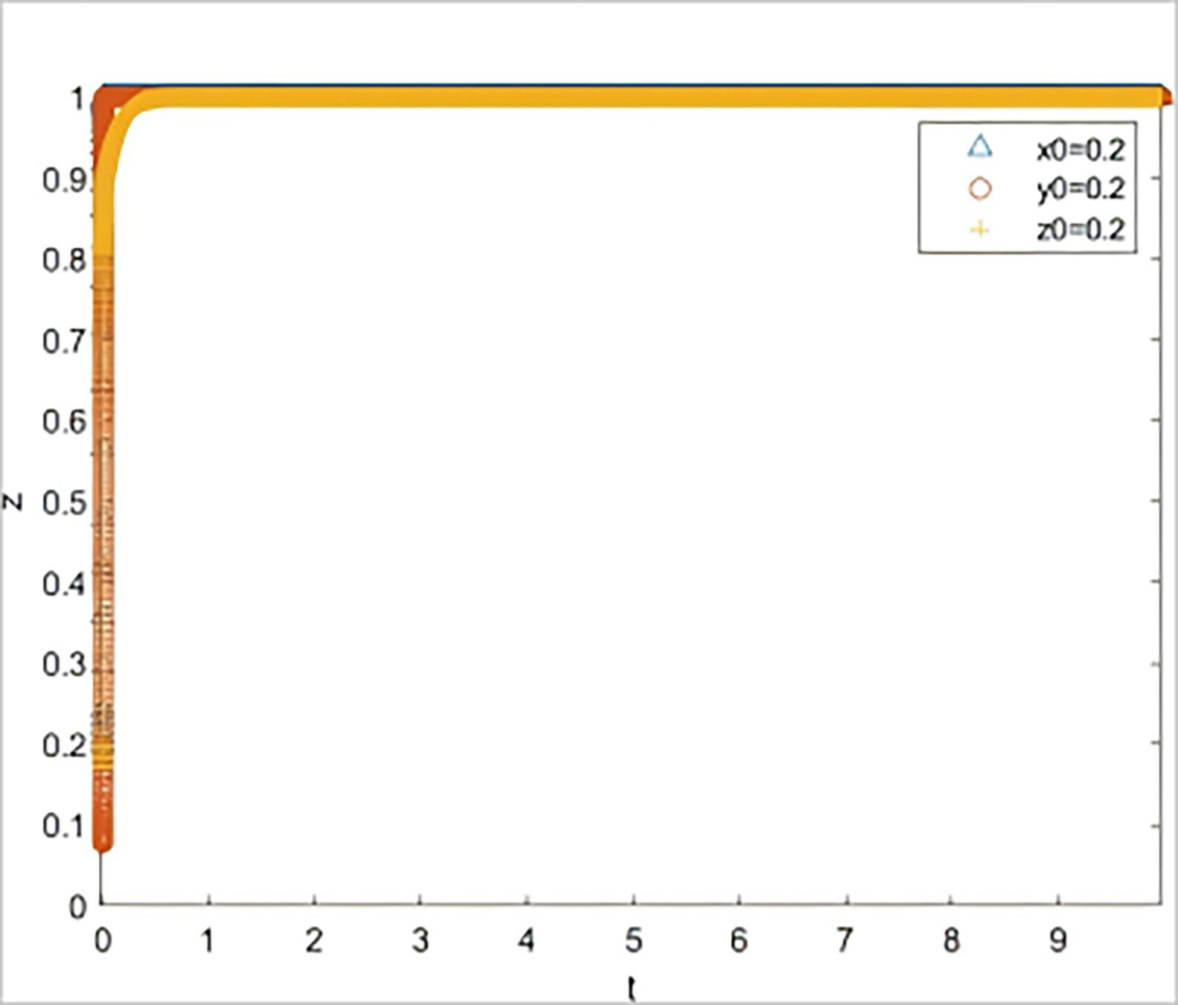
The impact on system evolution when the bank penalty is reduced (*C*_*f1*_ *= 10*).

With all other scenario conditions held constant, it is assumed that C_f2_ remains at 30, while C_f1_ is reduced to 20. The simulation results, as shown in Fig 10, indicate no change in the system’s operation. This suggests that, compared to guarantee institutions, banks are also not sensitive to the government’s punishments. Consequently, the government can appropriately reduce its supervision of banks’ responsibility fulfillment and focus more regulatory efforts on guarantee institutions.

In summary, comparing the simulation results of the four experimental scenarios reveals that guarantee institutions are highly sensitive to both the rewards and punishments from the government, due to the fact that they bear more risk costs compared to banks. On one hand, they require support from the government to cover the high risks they face, and thus are more inclined to fulfill their responsibilities actively in order to gain more rewards. On the other hand, guarantee institutions are apprehensive about bearing high penalties for not fulfilling their responsibilities diligently, so if the penalties are reduced, the deterrent effect will be weakened.

In contrast to guarantee institutions, banks are not as sensitive to the rewards and punishments from the government. Therefore, the government should allocate more reward and regulatory costs to guarantee institutions, thereby more efficiently guiding both banks and guarantee institutions to fulfill their responsibilities diligently.

#### (3) Scenario simulation of the change in the cost-sharing ratio of banks and guarantee institutions when fulfilling their responsibilities

When banks and guarantors choose to fulfill their responsibilities, they work together to reduce risk and expand the multiplier of SME financing and guarantees. In an ideal scenario, the total cost incurred by these efforts is denoted by *C*_*s*_. When both subjects take their responsibilities seriously, they cooperate and share the cost. In the initial scenario, let the ratio of the bank and the guarantor in cost-sharing be *λ*_*2*_ *= 0*.*3*, *μ*_*2*_ *= 0*.*7* (*λ*_*2*_*+μ*_*2*_ *= 1*). This is in line with the reality that banks with much say bear fewer costs, and guarantee institutions with little say bear more. Other conditions remain unchanged: it is assumed that the bank intends to reduce its own performance and transfer costs to the guarantee agency (*λ*_*2*_ *= 0*.*1*, *μ*_*2*_ *= 0*.*9)*.

**Fig 11:**
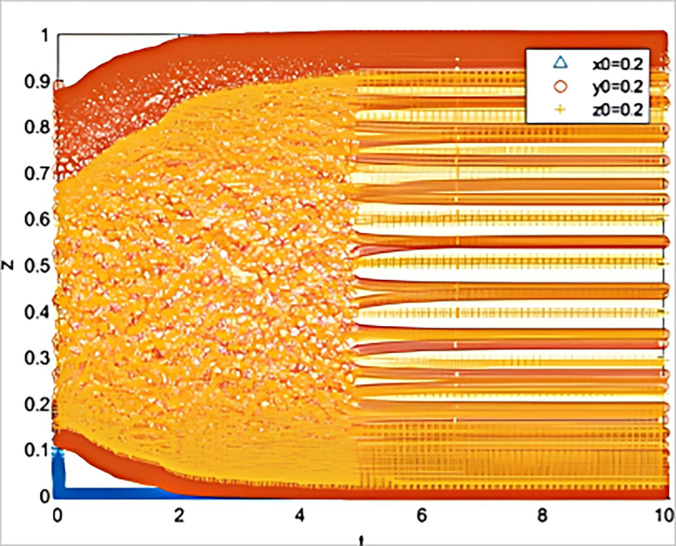
The impact on system evolution when the bank transfers costs to the guarantee agency(*λ*_*2*_ *= 0*.*1*, *μ*_*2*_ *= 0*.*9)*.

The simulation results, as shown in Fig 11, indicate that the system has entered an unstable state. This demonstrates that under conditions of high total costs, if banks shirk their responsibilities, all costs will be borne by the guarantee institutions. At this point, guarantee institutions may abandon active strategies due to excessively high costs, which in turn leads to a chain reaction, increasing the risk costs for banks and the government, thereby pushing the system into instability. Therefore, banks must take on their responsibilities and share part of the costs. This also indicates that the cost of fulfilling responsibilities between banks and guarantee institutions should be reasonably shared, especially avoiding the evasion of responsibilities by banks. It is necessary to mobilize the enthusiasm of both banks and guarantee institutions to encourage both parties to actively fulfill their responsibilities.

## 6. Discussion

### 6.1 Research results

This paper constructs a tripartite cooperative evolutionary game model for the operation of the GBGI model and explores the role transformation and measures of the government in the operation of the GBGI model. The study suggests that on the basis of existing functions such as credit endorsement, risk sharing, and information resource allocation, the government can further adopt strategic guidance measures. These include leveraging the advantage of grassroots information acquisition to provide high-quality risk information of SMEs for banks and guarantee institutions, assisting banks and guarantee institutions in accurately identifying, screening, and controlling the risks of SME customers; supervising and discerning the performance of banks and guarantee institutions, adopting differentiated reward and punishment measures; encouraging banks and guarantee institutions to share the cost of risk identification, and to carry out sustainable cooperation based on high-quality risk information; and encouraging banks to recognize the active responsibility of guarantee institutions, etc. Ultimately, it guides the sustainable and optimized development of the GBGI model. The specific conclusions are as follows:

1. In terms of the strategies of the three main bodies in the GBGI model, the benefits gained, default losses undertaken, and guidance costs incurred under the government’s two types of guidance strategies affect its behavioral choices. Similarly, the benefits, default losses, punishment costs, and responsibility costs under the two types of responsibility strategies of banks and guarantee institutions also influence behavioral choices. Furthermore, factors such as the quality of risk information, default risk and losses, reward benefits and punishment costs, and the sharing ratio of responsibility costs are all related to the behavioral choices of each main body, and the combination of these factors will bring about changes in the overall system scenario.
2. In terms of the operational state of the GBGI model, the quality of the SME risk information provided by the government to banks and guarantee institutions is one of the key factors affecting the system’s default rate, which in turn further affects the active cooperation behavior of the three parties and the sustainability of the system’s operation. Therefore, when implementing strategic guidance, the government must not only provide risk information of SME customers but also ensure the accuracy and reliability of the risk information. This can not only reduce the system’s default rate but also share the responsibility costs of banks and guarantee institutions, which is a necessary guiding measure to achieve the sustainable development of the GBGI model and to enter a virtuous cycle.
3. The serious responsibility behavior of banks and guarantee institutions in accurately identifying the risks of SMEs based on the high-quality risk information provided by the government is the second key factor affecting the system’s default rate. Therefore, the government needs to implement differentiated reward and punishment strategies, rewarding banks and guarantee institutions that take their responsibilities seriously, and punishing those that are passive, thereby motivating more banks and guarantee institutions to adopt proactive risk control behaviors and reduce the overall risk of the system.
4. Guarantee institutions are highly sensitive to both the rewards and punishments from the government, whereas banks are comparatively less sensitive. Therefore, the government should allocate more regulatory and reward costs to guarantee institutions, by increasing the disparity in rewards and punishments for different behaviors of guarantee institutions, to motivate them to fulfill their responsibilities diligently, and to lead banks into a state of serious responsibility. This can save the government’s guidance costs and more efficiently promote the system into a virtuous cycle.
5. If the government wants to mobilize the enthusiasm of banks and guarantee institutions to fulfill their responsibilities seriously, achieving the sharing of responsibility costs is one of the important driving forces. If the cost of fulfilling responsibilities is too high, the behavior of the main bodies will evolve in the wrong direction. To reduce and save costs, firstly, the government’s high-quality risk information remains key, which can eliminate part of the costs in terms of manpower, materials, and finances for banks and guarantee institutions in searching for information. Secondly, banks and guarantee institutions can reduce their responsibility costs by cooperating with each other to achieve a win-win situation. The idea of banks, which hold the initiative, wanting to take a free ride, hoping that guarantee institutions pay more while they only benefit themselves, is not sustainable. Therefore, the government needs to guide all three parties to jointly build a long-term mechanism for a good cost-sharing. At the same time, the government should also encourage banks and guarantee institutions to jointly build a long-term cooperation mechanism, encourage banks to give more recognition to guarantee institutions that take their responsibilities seriously, so that guarantee institutions can gain more business growth benefits, reputation benefits, etc., in long-term cooperation. This can not only further reduce the government’s guidance costs but also effectively promote the GBGI model to develop more efficiently and stably.

### 6.2 Theoretical implications

This investigation uncovers new insights into the fabric of strategic economic behavior and propels forward the theoretical understanding of cooperative dynamics within financial ecosystems. At the crux of this analysis lies the elucidation of a novel tripartite cooperation model which demonstrates the intricate interplay between government entities, financial institutions, and guarantee agencies. The introduction and application of this paradigm-shifting model offer a thorough conceptual framework that deepens the discourse surrounding the mitigation of systemic risks and amplifies the dialogue on sustainable financial practices.

Through this study, the importance of the quality of risk information has been revealed. We discussed that the government’s provision of high-quality risk information for SMEs is an important way to resolve the risk information asymmetry between banks, guarantee institutions, and SMEs, and it is also a crucial factor in mobilizing the enthusiasm of banks and guarantee institutions to leverage their own advantages in identifying the risks of SMEs.

The provision of high-quality risk information by the government is pointed out as the key to maintaining and advancing a sound financial infrastructure. At the same time, the government’s strategic and differentiated rewards and punishments are also key to stimulating the enthusiasm of banks and guarantee institutions. In addition, this study’s dissection of the cost-sharing mechanism reveals an incentive strategy that is consistent with the principle of collective responsibility, promoting stakeholders towards cost-efficient and fair financial burden distribution.

### 6.3 Practical implications

Our findings possess transformative potential for policymaking, delineating clear directive paths for governmental intervention in SME financing. By charting a systematic risk identification framework and advocating differentiated reward strategies, this paper supplies actionable insights that can be instrumental in overhauling existing policy frameworks. It also underscores the imperative of fostering enduring partnerships between banks and guarantee institutions. By favoring and incentivizing responsible conduct within these collaborations, governments can solidify financial stability and engender a conducive environment for SME growth.

An articulation of a sustainable development path for SME financing systems manifests in this work. It advocates a measured yet forward-thinking approach that places a premium on the integrity of risk information and the efficacy of cost-sharing mechanics. These components stand as critical fulcrums in achieving a financial paradigm that not merely survives but thrives.

### 6.4 Limitations and future research

While this study carves new territory in financial cooperation research, it acknowledges certain limitations that may offer fertile ground for future inquiries. Predominantly relying on a mathematical and conceptual lens, the study lacks empirical substantiation from real-world data. It scopes its focus narrowly on SME financing guarantee systems, without probing into the broader financial market landscape and the multiplicity of impacting governmental policy dimensions.

Subsequent research could undertake the mantle of validating the theoretical constructs presented herein through rigorous empirical analysis, drawing from granular data sets across multiple industries and regions. Investigations that scrutinize the influence of diverse factors on sustainable development or the specific impact of government policies can serve to refine and augment the theoretical groundwork laid by this study, accelerating the evolution of financial scholarship and practice.

## Supporting information

S1 File (DOCX)
