# Supplementary material for: The role of government in helping SMEs to access finance: An evolutionary game modeling and simulation approach
Source: PLoS One. 2024 Dec 27;19(12):e0315941. doi: 10.1371/journal.pone.0315941 (PMC11676567; doi:10.1371/journal.pone.0315941)
Supplement: S1 File — (DOCX) [file pone.0315941.s001.docx]

clear;

clc;

[t,y]=ode45(@xyz_function,[0,1],[0.2,0.2,0.2]);

plot3(y(:,1),y(:,2),y(:,3));

str=sprintf('演化示意图 \n x0=0.2,y0=0.2,z0=0.2');

title(str)

xlabel('x')

ylabel('y')

zlabel('z')

xlim([0 1])

ylim([0 1])

zlim([0 1.001])

grid on

set(gca,'GridLineStyle',':','GridColor','k','GridAlpha',1)

%axis square

clear;

clc;

[t,y]=ode45(@xyz_function,[0,10],[0.2,0.2,0.2]);

plot(t,y(:,1),'^',t,y(:,2),'o',t,y(:,3),'+');

str=sprintf('演化示意图 \n');

title(str)

ylim([0,1])

xlabel('t')

ylabel('z')

legend('x0=0.2','y0=0.2','z0=0.2');

%plot3(y(:,1),y(:,2),y(:,3));

%eq2.m文件

%描述微分方程组

function dy=xyz_function(t,y)

%说明微分变量是二维的，令y(1)=x,y(2)=y,y(3)=z

dy=zeros(3,1);

H=300;

Q=2;

Cg1=200;

Cg2=200;

k=10000;

R1=5.5%;

R2=3%;

n0=1.5;

n1=5;

n2=2.5;

A1=0.2;

A2=0.2;

A3=0.6;

V1=0.017;

V2=0.057;

V3=0.057;

V4=0.3;

V5=0.25;

V6=0.25;

V7=0.25;

λ1=1;

λ2=0.3;

μ1=1;

μ2=0.7;

Cs=200;

Ck=50;

Cf1=30;

Cf2=30;

Zb=40;

Zg=40;

w=40

dy(1)=y(1)*(1-y(1))*(Cg1-Cg2+H-k*n0*(A1+Q)*(V2-V6)*y(2)+k*(-n0*(A1+Q)*(V3-V7)+(-n2*Q+n1*(Q-(A1+Q)*V1)+n2*(A1+Q)*V5+n0*(A1+Q)*(V2+V3-V6-V7))*y(2))*y(3));

dy(2)=y(2)*(1-y(2))*(-C4+k*R1*(n0*(V4+V6*(-1+y(1))-V2*y(1))+(n2*(-1+V5)*(-1+y(1))-n1*(-1+V1)*y(1)+n0*(-1-V4+V6+V7+(V2+V3)*y(1)-(V6+V7)*y(1)))*y(3))+A2*k*(n1*(V5*(-1+y(1))-V1*y(1))*y(3)+n0*(V4+V6*(-1+y(1))-V2*y(1)+(-V4+V6+V7+(V2+V3)*y(1)-(V6+V7)*y(1))*y(3)))+y(1)*(Cf1+Zb)+Cs*(-1+y(3))*a1-Cs*y(3)*a2);

dy(3)=y(3)*(1-y(3))*(Cf2*y(1)+w*y(1)*y(2)+k*R2*((n2*(-1+V5)*(-1+y(1))-n1*(-1+V1)*y(1))*y(2)+n0*(V4-V7-V3*y(1)+V7*y(1)+(-1-V4+V6+V7+(V2+V3)*y(1)-(V6+V7)*y(1))*y(2)))+A3*k*(n2*V5*(-1+y(1))*y(2)-n1*V1*y(1)*y(2)+n0*(V4+V7*(-1+y(1))-V3*y(1)+(-V4+V6+V7+(V2+V3)*y(1)-(V6+V7)*y(1))*y(2)))+y(1)*Zg-y(2)*Zg+w*y(2)*Zg+y(1)*y(2)*Zg-w*y(1)*y(2)*Zg-Cs*b1+Cs*y(2)*b1-Cs*y(2)*b2);

End

clear;

clc;

[t,y]=ode45(@xyz_function,[0,10],[0.2,0.2,0.2]);

plot(t,y(:,1),'^',t,y(:,2),'o',t,y(:,3),'+');

str=sprintf('演化示意图 \n');

title(str)

ylim([0,1])

xlabel('t')

ylabel('z')

legend('x0=0.2','y0=0.2','z0=0.2');

%plot3(y(:,1),y(:,2),y(:,3));
